# Towards the Pharmacological Validation and Phytochemical Profiling of the Decoction and Maceration of *Bruguiera gymnorhiza* (L.) Lam.—A Traditionally Used Medicinal Halophyte

**DOI:** 10.3390/molecules27062000

**Published:** 2022-03-20

**Authors:** Nabeelah Bibi Sadeer, Kouadio Ibrahime Sinan, Zoltán Cziáky, József Jekő, Gokhan Zengin, Rajesh Jeewon, Hassan H. Abdallah, Yusra AlDhaheri, Ali H. Eid, Mohamad Fawzi Mahomoodally

**Affiliations:** 1 Department of Health Sciences, Faculty of Medicine and Health Sciences, University of Mauritius, Réduit 230, Mauritius; nabeelah.sadeer1@umail.uom.ac.mu (N.B.S.); r.jeewon@uom.ac.mu (R.J.); 2 Department of Biology, Science Faculty, Selcuk University, Konya 42130, Turkey; sinankouadio@gmail.com; 3 Agricultural and Molecular Research and Service Institute, University of Nyíregyháza, 001 Nyíregyháza, Hungary; cziaky.zoltan@nye.hu (Z.C.); jjozsi@gmail.com (J.J.); 4 Chemistry Department, College of Education, Salahaddin University-Erbil, Erbil 44001, Iraq; hassan.abdullah@su.edu.krd; 5 Department of Biology, College of Science, United Arab Emirates University, Al Ain P.O. Box 15551, United Arab Emirates; Yusra.aldhaheri@uaeu.ac.ae; 6 Department of Basic Medical Sciences, College of Medicine, QU Health, Qatar University, Doha 2713, Qatar

**Keywords:** *Bruguiera gymnorhiza*, diabetes, antioxidant, enzymes, docking, multivariate analysis

## Abstract

Decoctions (leaves and roots) of *Bruguiera gymnorhiza* (L.) Lam. are traditionally used against diabetes in many countries, including Mauritius. This study endeavoured to evaluate the inhibitory potential of leaves, roots, twigs and fruits extracts (decoction and maceration) of *B. gymnorhiza* against key enzymes relevant to diabetes. Considering complications related to diabetes, other clinical enzymes, namely, acetylcholinesterase (AChE), butyrylcholinesterase (BChE), tyrosinase, elastase and pancreatic lipase, were used. Identification of compounds was carried out using ultra-high-performance liquid chromatography/electrospray ionization tandem mass spectrometry (UHPLC-ESI-MS/MS). Antioxidant capacities were assessed using DPPH, ABTS, FRAP, CUPRAC, phosphomolybdenum, metal chelating. The relationship between mode of extraction, plant parts and biological activities was determined using multivariate analysis. Macerated fruits, rich in phytochemicals (phenolic, flavanol, tannin, and triterpenoid), exhibited substantially high antioxidant capacities related to radical scavenging (DPPH: 547.75 ± 10.99 and ABTS: 439.59 ± 19.13 mg TE/g, respectively) and reducing potential (CUPRAC: 956.04 ± 11.90 and FRAP: 577.26 ± 4.55 mg TE/g, respectively). Additionally, the same extract significantly depressed AChE and BChE (3.75 ± 0.03 and 2.19 ± 0.13 mg GALAE/g, respectively), tyrosinase (147.01 ± 0.78 mg KAE/g), elastase (3.14 ± 0.08 mg OE/g) and amylase (1.22 ± 0.01 mmol ACAE/g) enzymatic activities. Phytochemical results confirmed the presence of 119 compounds in all maceration and 163 compounds in all decoction samples. The screening also revealed important compounds in the extracts, namely, quinic acid, brugierol, bruguierol A, epigallocatechin, chlorogenic acid, to name a few. Multivariate analysis reported that the plant parts of *B. gymnorhiza* greatly influenced the observed biological activities in contrast to the types of extraction methods employed. Docking calculations have supported the findings of the experimental part through the high binding affinity and strong interactions of some compounds against tyrosinase, AChE, BChE and elastase enzymes. The decocted root and leaf of *B. gymnorhiza* showed low to moderate antidiabetic activity, thereby partially supporting its traditional uses in the management of diabetes. However, the fruit, the most active organ, can be used as a diet supplement to reduce the risk of diabetes complications after evaluating its cytotoxic effects.

## 1. Introduction

For centuries, our ancestors were largely dependent on home remedies and natural treatments to assuage sufferings mainly due to poverty and a lack of advanced technological resources. Similarly, phytomedicine is not recent in Mauritius but dates back more than 300 years [1]. A total of 561 plants are recorded as medicinally relevant in Mauritius with 52 of them traditionally used to manage diabetes mellitus (DM) type 2 and its related complications [1,2]. However, an insufficient amount of consolidated literature is noticed to substantiate such a presumption, leaving some of these plants unvalidated. One such underexplored plant is a mangrove species named *Bruguiera gymnorhiza* (L.) Lam. (*B. gymnorhiza*). Despite decoctions (leaves and roots) of this plant being the most commonly used mangrove species to control DM type 2 both globally and locally [3], it lacks pharmacological validation. In our previous paper, the organic extracts of *B. gymnorhiza* were screened [4]. However, in this study, preparation of aqueous extracts was a simpler, economical and eco-friendly alternative to the organic extracts. Furthermore, as far as our literature search could reach, no study has been conducted yet in Mauritius to confirm if the decoctions of *B. gymnorhiza* indeed have an anti-diabetic effect. Since people still follow this traditional practice, it is high time to substantiate such a presumption. Thus, the present study aimed at embarking on research to fill this niche by pharmacologically validating *B. gymnorhiza.*

The diabetes landscape has seen unparalleled changes during recent years. On the one hand, there has been a remarkable and consistent improvement in the clinical care: namely, development of new phytoagents for diabetes management, consolidated technical advances in glycaemic monitoring or new dietary approaches have been established. On the other hand, these advanced developments and treatments are insufficient as yet to overcome this global burden of prediabetes and the diabetes epidemic, since the number of people diagnosed with DM has skyrocketed in recent years [5]. For instance, in the year 2017, 425 million people aged between 20–79 were diagnosed with DM type 2, and it is the third most common disease among children after asthma and epilepsy [6,7]. On this note, it can be said that little is known on the mechanism of this alarming global health threat and thus, the quest for effective and novel antidiabetic drugs should be an on-going challenge and a continuing need, which is the focal point of this study.

Mangroves are considered an important medicinal plant based on their salient history in the medical lore. Apart from DM, the different plant parts of *B. gymnorhiza* can also be used against diarrhoea, fever, eye diseases, haemorrhage, liver disorders, shingles, or to treat burns, stings from toxic lagoon fishes and even to remove intestinal worms [3,8,9,10,11]. Herbs and natural products are rich in medicinal ingredients, and their therapeutic healing properties have been known since ancient times. Similarly, as reported in one of our comprehensive reviews, mangroves have huge potential for a wide array of medicinal products and drug discovery to prevent and treat many diseases as they can yield terpenoids, tannins, steroids, alkaloids, flavonoids, and saponins were the main classes of phytochemicals [12]. For instance, galantamine, tacrine, rivastigmine and donepezil are some of the common AChE inhibitors that moderate the deficiency of cholinergic neurons in an AD patient, by slowing the degradation of acetylcholine through AChE inhibition. Unfortunately, these drugs have been reported to be associated with some side effects such as nausea, diarrhoea and hepatotoxicity [13].

Existing literature revealed that the methanolic leaf, bark and root extracts of *B. gymnorhiza* significantly quenched DPPH radicals with IC50 values of 2052.20, 254.69 and 1532.71 µg of dry material, respectively [14]. Furthermore, a few research groups conducted in vivo analysis by screening *B. gymnorhiza* for its antinociceptive and antihyperlipidemic effects. Results showed that the methanolic leaf extract significantly inhibited writhing in acetic-acid induced mice [8]. In terms of antihyperlipidemic activity, the ethanolic root extract significantly decreases the level of total cholesterol, triglycerides, low-density lipoprotein (LDL) and very low-density lipoprotein (VLDL) [15]. On the other hand, Uddin and co-workers reported that the aqueous extract showed an averaged cytotoxic activity against breast cancer cell MDA-MB-435S with IC50 value of 1.38 mg/mL [16]. However, these results are unsystematic, fragmented and do not provide a clear conspectus on the pharmacological aspects related to diabetes and its associated complications of *B. gymnorhiza*. Furthermore, an absence of detailed phytochemical profiling is noticed in existing literatures which markedly limits our understanding of its biological abilities.

Therefore, this lack of knowledge prompted the compilation of the present paper. We aimed at exploring the antidiabetic properties of *B. gymnorhiza* via inhibition of key enzymes linked to diabetes and related complications, namely, α-amylase, α-glucosidase and pancreatic lipase. Given recent association of diabetes with neurodegenerative disorders stating that DM type 2 is an accelerator and risk factor for dementia [17], we also aimed at investigating the anti-acetyl- and butyryl-cholinesterase activities. Furthermore, several clinical studies reported that 79.2% diabetic patients developed cutaneous disorders during the progression of the disease [18]; ultimately, we also screened *B. gymnorhiza* against tyrosinase and elastase enzymes since they are the two most important enzymes linked to skin problems. In addition, the phytoconstituents were characterized using ultra high-performance liquid chromatography/electrospray ionization tandem mass spectrometry (UHPLC-ESI-MS/MS) and multivariate and docking analysis were conducted to obtain more information on the collected scientific data.

## 2. Results

### 2.1. Bioactive Compounds

Phytochemicals, also known as secondary metabolites, are bioactive compounds from plants and are the derivation of most medicines currently available on the shelf of pharmacies. Indeed, as an ample evidence, a survey demonstrated that 77% of antibiotics and 547 drugs approved by the Food and Drug Administration (FDA) by the end of 2013 originated from natural products [19]. Natural products play a crucial role in drug development and thus, screening plants with the aim to identify significant active constituents can be considered as a first step towards the development of more effective drugs against a wider spectrum of diseases. Joining this ongoing challenge, the present study aimed at screening the different extracts prepared from different parts of *B. gymnorhiza* for their bioactive compounds.

As a first step, the prepared extracts were screened for a panel of bioactive components, namely, phenolic acid, phenolic, flavonoid, flavanol, condensed tannin and triterpenoid. The data collected are presented in Table 1. The observation from the present study showed that the fruit extract prepared via maceration was found to be richer in bio compounds in terms of phenolic, flavanol, tannin and triterpenoid contents (185.33 ± 1.03 mg GAE/g, 13.84 ± 0.16 mg CE/g, 97.85 ± 4.41 mg CE/g and 36.84 ± 2.30 mg OAE/g, respectively). However, fruit extract prepared through decoction did not yield a significant amount of phytoconstituents. The reason behind this can be linked with the association of heat in the extraction techniques applied. The thermolabile compounds present in fruit may have decomposed in decoction while staying stable in maceration which explains the difference in the amount of bio compounds quantified (Table 1). Nonetheless, it is noteworthy to point out that ANOVA did not reveal any statistical difference between root decoction and macerated fruit in terms of the amount of flavanol and tannin quantified. For instance, the root decoction possessed 13.38 ± 0.14 and 90.49 ± 5.32 mg CE/g of flavanol and tannin, respectively (Table 1). Another important factor to be considered in this study is the usage of four plant parts. Table 1 demonstrates that leaves and twigs yielded the least amount of phytoconstituents which consequently resulted in weak biological activities irrespective of the mode of extraction used.

By means of standard chemical tests, the quantitative analysis of the phytocomponents present in leaves, roots, twigs and fruits of *B. gymnorhiza* was only an estimate and limited to a set of bio compounds that we selected to quantify: namely, phenolic acid, phenolic, flavonoid, flavanol, condensed tannin and triterpenoid. However, it is possible that the tested extracts might possessed other class of phytochemicals. Therefore, to have an accurate overview on the set of phytochemicals present in the different plant parts, we conducted a thorough chemical characterisation using ultra high-performance liquid chromatography/electrospray ionization tandem mass spectrometry (UHPLC-ESI-MS/MS). Figures S1–S16 show all chromatograms of UHPLC-ESI-MS/MS of all extracts (Supplementary Materials).

Structural identification and characterization were carried out on the comparisons of their chromatographic and ESI-MS/MS data (retention time, exact mass and fragmentation pathway) with the corresponding standards and data reported in the previous literature and our recently published works. Phenolic acids, flavone aglycones, flavonoid glycosides, triterpenoids were identified in the extracts, but their number was different. Chemical composition of extracts showed wide variability according to the plant parts and methods of extraction used. There were 119 components in the maceration and 163 components in the decoction samples (Table 2, Table 3, Table 4 and Table 5). For instance, a total of 27 compounds were found in common in the fruit extracts followed by leaf with 19, twig with 8 and root with 39 compounds, irrespective of the methods of extraction used. The details results are given in Tables S1–S8.

### 2.2. Antioxidant Assays

Diabetes mellitus (DM) is highly acknowledged to impair the antioxidant defensive system of the body which, as a result, exposes the body to a condition known as oxidative stress. This physiological stress results from an imbalance between free radicals and antioxidants which consequently leads to a multitude of other pathological cases such as cancer, inflammation, cardiovascular and neurodegenerative diseases [20,21,22,23]. It is mentioned in a review entitled ‘Antioxidants and diabetes’ that hyperglycaemia promotes the auto-oxidation of intracellular glucose in the presence of transition metals forming an excessive amount of free radicals [24], which consequently lead to oxidative stress. Indeed, a bevy of existing literature supports the fact that the antioxidant defences of diabetic patients are weak, and a series of conditions are usually reported in diabetics such as low free-radical scavenging activity, oxidation of plasma and low capacity of antioxidant enzymes, particularly catalase, superoxide dismutase, glutathione peroxidase [24]. However, recent studies demonstrated that the intake of antioxidants may stymie DM type 2 by reducing oxidative stress and improving insulin sensitivity [21,25,26]. However, the efficacy of the current drugs is hampered by their discouraging side effects. As a result, natural agents from plants are preferred and became an alternative target to search for novel antioxidant and antidiabetic agents based on their traditional use. Taking into consideration that *B. gymnorhiza* is the most commonly used mangrove plant against diabetes, it is indeed a matter of great interest to intensify our research on its antioxidant capacity as summarised in Table 6.

**Figure 1:**
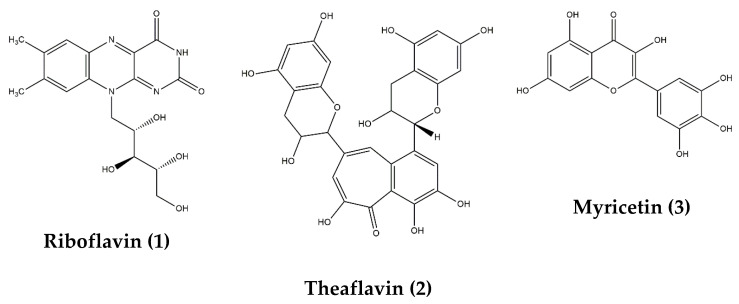
Chemical structures of compounds **1**–**3** isolated from maceration of *B. gymnorhiza* fruit but reported absent in decoction.

The antioxidant assays conducted herein followed different mechanisms; thus, we screened our samples for their antioxidant activities from different perspectives. From Table 6, it can be seen that the macerated fruit extract displayed substantially high antioxidant capacity particularly in terms of copper (II) reduction with CUPRAC assay followed by decocted root and macerated root extracts. This also coincides with our other analyses whereby these extracts yielded a high amount of phytochemicals (Table 6). Furthermore, through UHPLC-ESI-MS/MS, we identified the presence of various phytoconstituents in the maceration of *B. gymnorhiza* fruit which are known to possess potent antioxidant properties, namely, riboflavin (**1**), theaflavin (**2**) and myricetin (**3**) (Table 6, Figure 1). Riboflavin, also known as vitamin B_2_, is an antioxidant nutrient possessing preventive ability against lipid peroxidation, myricetin is a flavonoid derivative known for both its strong antioxidant property and nutraceutical value, while theaflavin is an effective antioxidant flavanol and potent antibacterial agent [27,28,29]. Thus, these phytoantioxidants could have acted synergistically, explaining our results of the antioxidant screening (Table 6).

**Figure 2:**
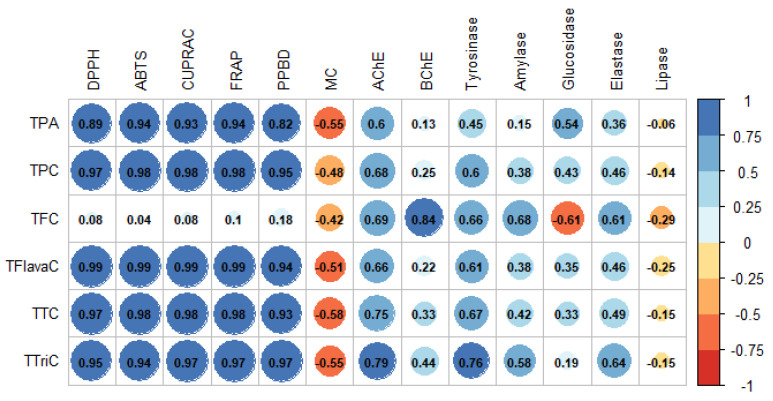
Pearson correlation between bio compounds quantified and biological activities (*p* < 0.05). Abbreviations: TPA: total phenolic acid; TPC: total phenolic content; TFC: total flavonoid content; TFlavaC: total flavanol content; TTC: total condensed tannin content; TTriC: total triterpenoid content.

To investigate the relationship between bio-compounds and bioactivities (antioxidant and enzymatic inhibitory assays), Pearson correlation analysis was done. The higher the Pearson correlation coefficient (R), the stronger the positive influence of a given compound on the given antioxidant and enzymatic properties [30]. In this study, a positive correlation was observed between most bio-compounds with their respective biological activities (Figure 2). A strong positive correlation with R coefficient greater than 0.8 was revealed between antioxidant assays, except metal chelating, and bio-compounds, except flavonoid. However, weaker R values were observed with enzymatic inhibition assays (−0.61 ≤ R ≤ 0.84) (Figure 2). Thus, it can be extrapolated that the respective bio-compounds had a stronger influence on antioxidant assays in contrast to the enzymatic assays. Although flavonoids are known to possess significant antioxidant and chelating properties [31,32], our data do not corroborate this fact. Instead, correlation analysis demonstrated a weak interrelationship between flavonoids and all antioxidant assays. Nevertheless, flavanols, being a subgroup of flavonoids, showed the strongest Pearson correlation coefficient ranging from 0.94 to 0.99 with most antioxidant assays except metal chelating. Flavanols are a class of flavonoids possessing a ketone group [33]. Since the interaction between flavanols and flavonoids with their respective biological activities are significantly different, we presumed that the presence of the ketone group might be bringing that demarcation in results.

With respect to radical scavenging activities, results showed that the aqueous *B. gymnorhiza* fruit extract was the most potent DPPH radical scavenger (547.75 ± 10.99 mg TE/g). Although both DPPH and ABTS are radical scavenging assays, they displayed different results since the ABTS radical chromogen solubilized in both organic and aqueous solvents while DPPH is soluble only in organic solvents [34]. Indeed, our aqueous extracts displayed better results with ABTS assay in contrast to DPPH. Similarly, to DPPH and ABTS assays, aqueous fruit extracts prepared by maceration exhibited the most potent reducing potential in CUPRAC and FRAP assays (956.04 ± 11.90 and 577.26 ± 4.55 mg TE/g, respectively).

The total antioxidant capacity (TAC) was assessed using phosphomolybdenum assay. The maceration of *B. gymnorhiza* fruit with a value of 5.34 ± 0.24 mmol TE/g demonstrated the optimal activity followed by decoction root (3.70 ± 0.08 mmol TE/g). The macerated fruit was found to possess the highest amount of phenolic, flavanol, tannin and triterpenoid. Thus, TAC may be ascribed to the presence of these bio-compounds present. It is known that excess transition metal ions present may lead to the formation of free radicals in our biological system which consequently cause various diseases [35].

Metals are known to enhance the pathogenesis of DM since they are responsible for causing the auto-oxidation of glucose in the cells, as previously explained in this study [36]. Furthermore, a number of publications reported that an elevated level of iron in the blood causes the oxidation of many biomolecules, namely, lipids, nucleic acids and proteins, resulting in a decrease in the secretion of insulin from pancreatic β-cells which concomitantly increase insulin resistance contributing to the development of DM type 2 [36,37]. Thus, by scavenging/chelating, iron can prove to be prophylactic towards DM type 2; however, this hypothesis requires further studies. Since the objective of our research work is to scrutinize *B. gymnorhiza* for possible antioxidant and antidiabetic agents, we opted to screen the extracts for iron chelating activity. According to our results, macerated twig exhibited the highest iron chelating activity followed by decoction leaf (31.45 ± 1.95 and 23.69 ± 0.40 mg EDTAE/g, respectively). Bearing in mind that a decoction of leaf is usually consumed for managing diabetes, it can be said that the results obtained from the iron chelating assay support its traditional uses.

### 2.3. Enzymatic Inhibitory Effects

Herein, the different extracts of *B. gymnorhiza* were evaluated for their inhibitory potential against numerous enzymes related to DM type 2 together with its associated complications, viz., obesity, cutaneous manifestations and Alzheimer’s disease (AD). For this pharmacological validation, a panel of enzymes were chosen whereby α-amylase and α-glucosidase are used for DM type 2, AChE and BChE for AD, tyrosinase and elastase for cutaneous manifestations and pancreatic lipase for obesity.

The pancreas of diabetic patients can be sluggish to secrete insulin in response to a meal which consequently lead to a condition known as post-prandial hyperglycaemia (elevated blood glucose level). One way to prevent this condition is by hindering the breakdown of dietary polysaccharides into glucose in the gastrointestinal tract. Since α-amylase and α-glucosidase are digestive enzymes released in the intestine to digest carbohydrates into glucose when a meal is present in the digestive tract, inhibition of these enzymes will therefore help to reduce the amount of glucose absorbed into the body [38]. Data gathered herein showed that the extracts were more potent inhibitors of α-glucosidase compared to α-amylase. Although all extracts showed inhibition against α-amylase, several extracts were noted as ineffective against α-glucosidase (Table 7).

The most potent extracts in α-glucosidase inhibition demonstrated a weak inhibition against α-amylase. For instance, maceration root extract inhibited α-glucosidase with an acarbose equivalent value of 31.18 while α-amylase was inhibited with an equivalent value of 0.13. At the present time, development of antidiabetic agents having lower inhibitory effect against α-amylase but marked inhibition against α-glucosidase are mostly preferred [39]. Furthermore, phenolic compound is a major factor to be considered in enzymatic inhibitory effects. For instance, it is acknowledged that phenolic compounds showed evidence of pronounced α-glucosidase inhibition property but milder inhibitory action against α-amylase [40]. Thus, the significant α-glucosidase inhibitory property reported herein with maceration fruit and decoction root might be attributed to their phenolic composition (Table 1).

Diabetes mellitus should not be regarded as only a disorder caused by an abnormal glucose homeostasis but is also the outset of other diseases. For instance, a large majority of diabetics are prone to different types of cutaneous problems with diabetic dermopathy and xerosis reported as the most common followed by acanthosis nigricans [18]. Diabetic dermopathy are light brown, irregular patches formed on the skin, xerosis is a condition caused by a lack of moisture and elasticity in the skin [41,42,43] while acanthosis nigricans is a velvety brown hyperpigmentation usually located at flexion folds such as armpits [44]. Herein, we have selected two enzymes in relation to these diabetic skin disorders, namely, tyrosinase and elastase, to screen the extracts of *B. gymnorhiza*. Hyperpigmentation is caused by tyrosinase; thus, inhibition of tyrosinase activity can be a good therapeutic approach for treating cutaneous hyper pigmentary disorders such as diabetic dermopathy and acanthosis nigricans [45]. Polyphenols, namely, flavonoids and stilbenoids, are acknowledged to significantly impede tyrosinase activity. Our results demonstrated that maceration fruit was the most potent inhibitor with a kojic acid equivalent value of 147.01 followed by decoction fruit (101.93 ± 2.45 mg KAE/g). Inhibitory tyrosinase property is related to phenolic compounds, namely, quercetin, benzoic acid and their derivatives. It is stated that quercetin is a flavonoid recognised for its strong anti-melanogenesis activity [46,47]. Indeed, this statement is in agreement with our present work since UHPLC-ESI-MS/MS identified the presence of quercetin in the maceration of *B. gymnorhiza* fruit, and hence, it can be hypothesised that the presence of this flavonoid contributed to the strong anti-tyrosinase activity of that extract. In addition to tyrosinase, the extracts were screened for their elastase inhibitory activities since xerosis is a condition caused by a loss of elasticity in the skin of diabetics. Our results showed that both maceration and decoction fruit displayed strong inhibitory effects against elastase with catechin equivalent value of 3.14 and 2.76, respectively. Detailed phytochemical screening by UHPLC-ESI-MS/MS confirmed the presence of catechin in both extracts. On this note, it can be said that the strong elastase activity might be attributed to the compound catechin, since it was reported to possess significant inhibitory activity against elastase [48]. As a result, we suggest that the macerated fruit extract represents potential for use as a topical treatment against cutaneous disorders, particularly, diabetic dermopathy, acanthosis nigricans and xerosis.

Diabetes mellitus and obesity are interrelated, and studies claimed that insulin sensitivity is affected by body fat distribution. For instance, individuals whose body fat is distributed peripherally have better insulin sensitivity in contrast to individuals with centred body fat distribution [49,50]. It is reported that 90% of diabetics are attributable to excessive weight [51]. However, in terms of lipase inhibition, only decoction twig showed activity with an orlistat equivalent value of 13.81 while other extracts were ineffective. Hence, *B. gymnorhiza* can be excluded as a candidate for managing obesity.

Alzheimer’s disease (AD) is a progressive neurodegenerative disease affecting a large portion of the cerebral cortex and cognitive ability [52]. Biessels stated in his review published in *The Lancet* that people diagnosed with DM type 2 have a 50% risk of developing dementia in contrast to people without diabetes [53]. Additionally, a review compiled by Kandimalla et al. stated that several researchers have even termed AD as a type 3 diabetes due to the shared molecular and cellular features among DM type 1 and 2 and insulin resistance associated with memory deficits and cognitive decline [54]. Recently, a growing body of solid evidence supports the link between DM type 2 and neurodegeneration. For instance, hyperinsulinemia (a characteristic of DM type 2 as a consequence of insulin resistance) is known to reduce the level of insulin in the central nervous system via downregulation of insulin transport at the blood brain barrier which ultimately reduced insulin signalling (a neuronal functioning which facilitates learning and memory) and weakened glucose transport resulting in neurodegenerative disorders, namely, AD [55,56]. Furthermore, it is important to highlight that a few studies reported that diabetes treatment does not only regulate glucose level but also improves cognitive performance and prevents AD [55,57,58]. Indeed, a study reported that to re-establish the normal functioning of insulin signalling, cholinesterase inhibitors are given to AD patients since these inhibitors are known to increase concentration of acetylcholine at synapses, thus improving memory, learning, attention and mood [59]. The types of cholinesterase enzymes used herein are AChE and its ‘sister’ enzyme BChE since they are both actively involved in catalysing the dissociation of acetylcholine resulting in the discontinuation of synaptic transmissions [60]. As presented in Table 7, the results for AChE and BChE inhibition activities were both correlated. For example, both maceration and decoction fruit reported the most active extracts for AChE (3.75 ± 0.03 and 3.90 ± 0.14 mg GALAE/g, respectively) and BChE (2.19 ± 0.13 and 2.85 ± 0.74 mg GALAE/g, respectively). Interestingly, the extracts demonstrated relatively higher galantamine equivalent values against AChE in contrast to BChE, suggesting they were more potent AChE inhibitors. Phytochemical screening characterised the presence of sinaptic acid (**4**) in both macerated and decocted fruit; however, it was absent in other extracts. Mounting evidence has elucidated the potent anticholinesterase activity of this compound [61,62]. Consequently, it can be extrapolated that the presence of sinaptic acid in the most active extracts against cholinesterase contributed to the high cholinesterase activities. However, both maceration leaf and twig extracts were found ineffective against AChE and BChE enzymes.

### 2.4. Multivariate Analysis

**Figure 3:**
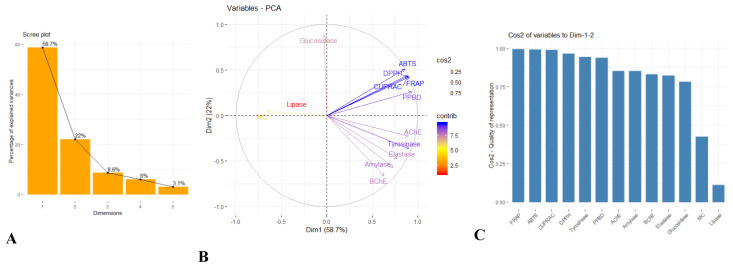
(**A**) Scree plot of percentage of explained variances; (**B**) correlation circle plot; (**C**) bar plot to visualize the quality of representation (cos2) on the correlation circle plot using the results of principal component analysis (PCA).

With reference to the scree plot of percentage of explained variances (Figure 3A) and according to the Kaiser rule, two principal components were retained, since both components summarize 80.7% of the total variance. This explains that the first two components retained the maximum information of the original data. Thus, the correlation circle plot is graphed to determine the correlation between variables based on these two retained principal components (PCs) or dimensions (Dims) (see Figure 3B). Herein, most of the variables were positively correlated since they were clustered together. For instance, the variables, namely, ABTS, DPPH, FRAP, CUPRAC and phosphomolybdenum (PPBD), displayed significantly positive correlation while glucosidase, lipase and metal chelating (MC) shared a negative correlation since they are positioned on the opposite site of the plot. Furthermore, the correlation circle plot shows uncorrelation between ABTS and BChE. The larger the value of the contribution, the more the variable contributes to the component. Herein, ABTS, FRAP and CUPRAC contribute most to PC2 (i.e., Dims 2) in contrast to the other variables. The quality of representation of the variables on a correlation circle plot is defined by the value of the squared cosine (cos2) (Figure 3C). In the present study, the sum of cos2 of most variables tends to one, showing a good quality of representation. However, since the cos2 values of MC and lipase were low (i.e., close to the centre), it can be said that these variables were not perfectly represented by the PCs or Dims.

**Figure 4:**
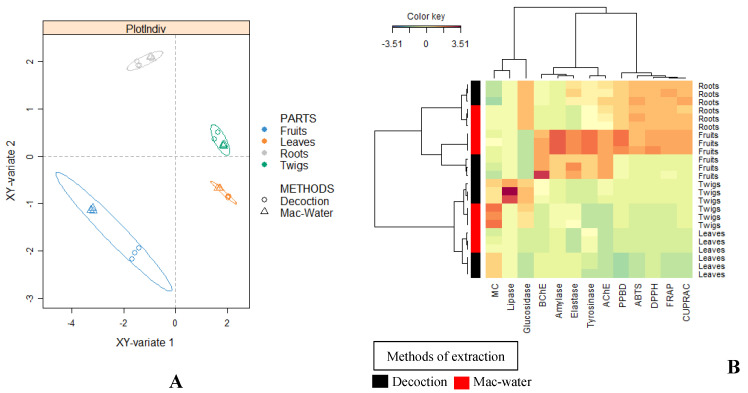
(**A**) Sparse PLS-DA score plot based on biological activities; (**B**) hierarchical cluster analysis (heatmap) based on biological activities.

As shown in Figure 4A, there was a good separation displaying four different groups in terms of the plant parts studied. The plot demonstrates that there is no clear-cut difference between the biological activities of maceration and decoction roots, twigs and leaves, since they are all clustered together. However, a slight difference is observed with maceration and decoction fruit. Thus, it can be said that the plant parts of *B. gymnorhiza* were a greater influencer on the observed biological activities in contrast to types of extraction methods employed. Likewise, the same results are reflected in the hierarchical clustering produced from a heatmap as shown in Figure 4B. From Figure 4, it can be said that it is the plant parts which significantly influenced the biological activities of the tested extracts and not the mode of extraction used.

**Figure 5:**
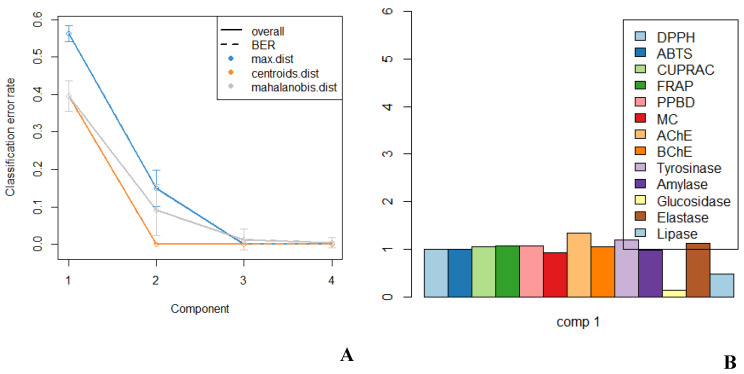
(**A**) Prediction model performance. (**B**) Variable importance in projection (VIP); biological activities with VIP greater than 1 were most relevant for discriminating the extracts.

The classification and prediction performance of sPLS-DA model was assessed by determining BER (balanced error rate) using both ‘maximum’ and ‘mahalanobis’ as prediction distances with 5-fold CV repeated 10 times, as illustrated in Figure 5A. The model shows a significant decrease in the classification error rate (i.e., an increase in classification performance) from one component to two components. Thus, the best performance (i.e., low error rate) seems to be achieved for ncomp = 2 with an error rate of approximately 0.1. Furthermore, the variable importance in projection (VIP) was graphed to select which variables contribute the most to the y variable explanation [63]. Herein, variables with VIP score > 1 were considered important, indicating that seven biological activities, namely, CUPRAC, FRAP, PPBD, AChE, BChE, tyrosinase and elastase were the main players in bringing variabilities in the tested extracts (see Figure 5B).

### 2.5. In Silico Docking

Docking is an essential tool to elucidate the potential biological activity of some dominant compounds with studied enzymes, theoretically. A list of potential inhibitors of tyrosinase, AChE, BChE and elastase enzymes were selected from the identified compounds in the extracts, and their chemical structures are shown in Table 8.

**Figure 6:**
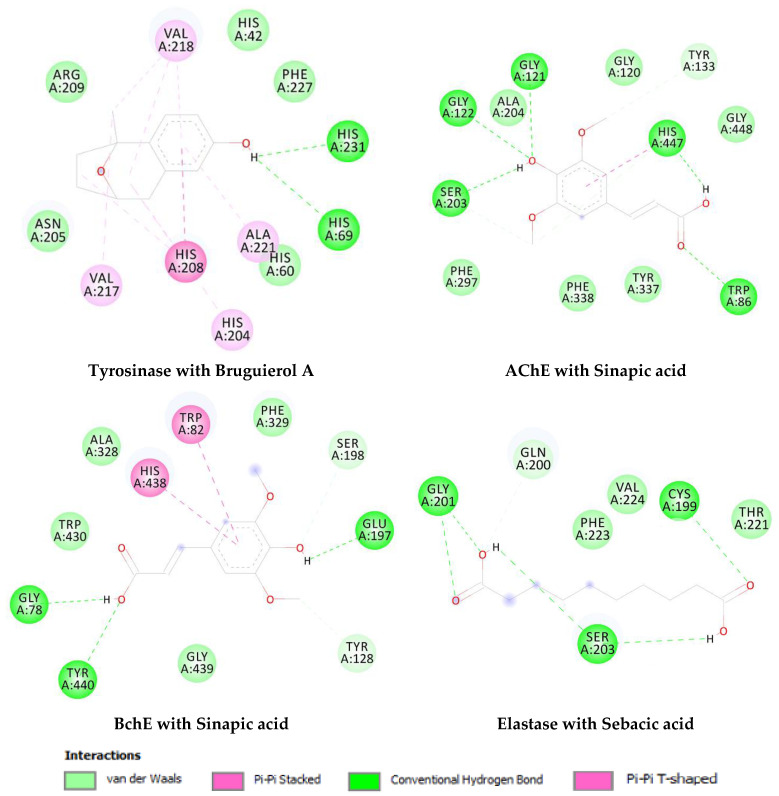
Enzyme-substrate interactions of the top docked compounds.

Following the docking calculations, parameters such as binding free energy and inhibition constants were determined and summarized in Table 9. Results showed that among the selected compounds, brugierol has the highest binding affinity for the tyrosinase enzyme. Sinapic acid has shown similar binding affinity with both AChE and BChE showing only a difference of 0.38 kcal/mol in their binding energies. Additionally, docking method showed that the ligand, sebacic acid, has a relatively high binding energy of −4.41 kcal/mol with elastase as the targeted enzyme. Figure 6 shows the nonbonding interactions of the docked compounds with the amino acid residues at the active site of the enzyme. Indeed, these interactions are based on the structure activity relationship of the chemical compound. Different interactions were formed between brugierol and tyrosinase enzyme; however, hydrogen bonds and pi-pi interactions were the strongest. Similar interactions were found for sinapic acid while in the case of sebacic acid, hydrogen bonds were the dominant interactions.

## 3. Materials and Methods

### 3.1. Collection of Plant Materials

The plant materials of *B. gymnorhiza*, namely, leaves, twigs, roots and fruits, were collected, by an authorized person from the government of Mauritius, along the coastline of Bambous Virieux in Mauritius (GPS: 20°20′13.88″ S; 57°45′54.99″ E) during the rainy summer season on 16 April 2018. The plant was authenticated by the botanist of the Mauritius Herbarium at the Mauritius Sugarcane Industry and Research Institute (MSIRI), Réduit, Mauritius. A voucher specimen bearing the number MAU 0029125 was deposited at the MSIRI herbarium. The use of the plant materials in this study complies with the international and national guidelines.

### 3.2. Extraction

The plant parts were carefully washed under running tap water to remove all surface debris and sands and allowed to dry in the shade. When a constant mass was noted, each dried plant part (leaves, roots, twigs, fruits) was powdered. The samples were prepared via two different types of extraction methods, namely, maceration (cold extraction) and decoction (hot extraction). Each plant part (50 g) was macerated in 500 mL distilled water while decoctions were prepared by allowing 50 g of plant parts to boil in 200 mL distilled water for 30 min. The extracts were filtered and concentrated in a rotary evaporator at a temperature set at 37 °C. The concentrated extracts were dried in a lyophilizer and stored at +4 °C in the dark until further analysis.

### 3.3. Profile of Bioactive Compounds

The total bioactive compounds, namely, total phenolic (TPC), flavonoid (TFC), phenolic acid (TPA), flavanol (TFlavC), condensed tannins (TTC) and triterpenoids (TTriC) were quantified using colorimetric methods as previously described [64,65,66]. The results were expressed as mg of reference compounds per g of dried extract. For instance, gallic acid was used for TPC, rutin for TFC, caffeic acid for TPA, catechin for TFlavC and TTC, oleanolic acid for TTriC.

A Dionex Ultimate 3000RS ultra high-performance liquid chromatography (UHPLC) equipment was used to determine the chemical contents of the *B. gymnorhiza* extracts. Before UHPLC analysis, the extracts were filtered using 0.22 μm PTFE syringe filters (Labex Ltd., Budapest, Hungary). The compounds were separated on a Thermo Accucore C18 column (100 mm × 2.1 mm i. d. 2.6 µm) thermostated at 25 °C (±1 °C). Water (A) and methanol (B) were employed as solvents, and both were acidified with 0.1% formic acid. The flow rate was kept constant at 0.2 mL/min. The column was connected to a Thermo Q Exactive Orbitrap mass spectrometer with electrospray ionization source (Thermo Scientific, Waltham, MA, USA). MS spectra were collected in both positive and negative-ion modes.

The following settings were used for the full scan: resolution of 70,000 (FWHM); collision energy of 35 (NCE); automated gain control (AGC) target of 3 × 10^6^; maximum injection duration of 100 ms; scan range of 100 to 1500 *m*/*z*. Resolution 35,000 (FWHM), AGC target 1 × 10^5^, maximum IT 50 ms, loup count 5, isolation window 1.0 *m*/*z* were used for fragmentation. The analysis took 70 min to complete.

Trace Finder 3.1 (Thermo Scientific, USA) software was applied for target screening. The compounds listed in Table 2, Table 3, Table 4 and Table 5 were identified on the basis of our previous published works or data found in literature using exact molecular mass, isotopic pattern and characteristic fragment ions. In every case, the exact molecular mass, isotopic pattern, characteristic fragment ions and retention time were used for the identification of the compounds which are marked that were confirmed by standards.

### 3.4. Determination of Antioxidant and Enzyme Inhibitory Effects

The bioactivities of the prepared extracts were screened for antioxidant capacities in terms of radical scavenging (2,2′-azino-bis(3-ethylbenzothiazoline-6-sulphonic acid (ABTS) and 2,2-diphenyl-1-picrylhydrazyl (DPPH)), reducing potential (ferric reducing antioxidant power (FRAP) and cupric reducing antioxidant capacity (CUPRAC)), total antioxidant capacity (phosphomolybdenum) and metal chelating (ferrozine method). Inhibitory assays against crucial clinical enzymes (α-amylase, α-glucosidase, pancreatic lipase, cholinesterase, tyrosinase and elastase) involved in diabetes and related complications were performed to highlight the enzymatic effects of the extracts. All procedures are described in our previous publications [67,68,69]. All results were expressed as equivalents of reference compounds per g of dried extract. Galantamine was used for cholinesterase (GALAE), kojic acid for tyrosinase (KAE), acarbose for α-amylase and α-glucosidase (ACAE), orlistat for pancreatic lipase (OE), catechin for elastase (CAE), Trolox for ABTS, DPPH, FRAP, CUPRAC and phosphomolybdenum (TE) and ethylenediaminetetraacetic acid (EDTA) for metal chelating (EDTAE).

### 3.5. Statistical Analyses

One-way analysis of variance (ANOVA) with post-hoc Tukey test was conducted to determine whether there are any statistically significant differences between the means of the tested extracts (*p* < 0.05). Pearson correlation and hierarchical cluster analysis (heatmap) were done to investigate the interaction between bio compounds and biological activities (antioxidant and enzymatic inhibition). Sparse Partial Least Squares (sPLS-DA) analysis was performed to assess the effect of the different plant parts used on the biological activities observed. The statistical procedures were achieved using R software v. 3.5.1 (R Core Team, Vienna, Austria).

### 3.6. In Silico Docking Calculations

**Figure 7:**
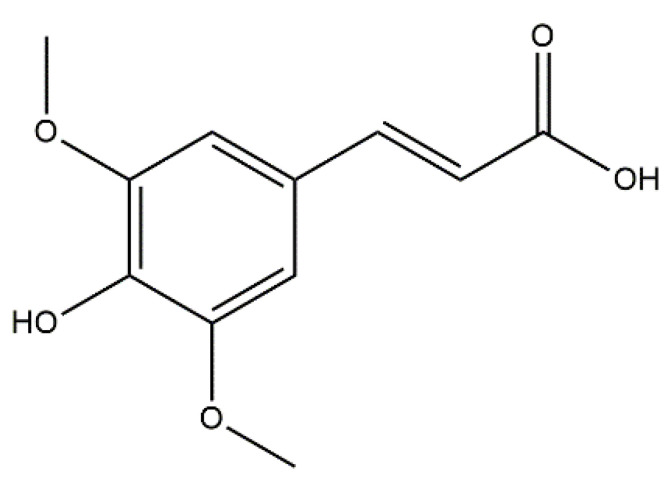
Chemical structure of sinapic acid (**4**).

In this study, brugierol, theaflavin, bruguierol A and sebacic acid were docked against tyrosinase enzyme. Sinapic acid (Figure 7) was docked against AChE and BChE enzymes and sebacic acid against elastase enzyme. The 2D structures of these bioactive compounds were downloaded from PubChem and ChemSpider online databases. The structures were then visualized and optimized using AM1 semiempirical method implemented in VegaZZ software and saved as mol2 format. On the other hand, the enzymes’ crystal structures of tyrosinase, AChE, BChE and elastase enzymes were downloaded from Protein Databank RCSB. The pdb code of the crystal structure of tyrosinase enzyme is 5I38 in which the enzyme was crystalized with kojic acid. The pdb code of the AChE enzyme is 4EY6 in which the enzyme was crystalized in complex with galantamine while the pdb code of the BChE is 1P0P in complex with butyrylthiocholine substrate. For the crystal structure of the elastase enzyme, the pdb code used for docking was 1HV7.

AutodockTools-1.5.6 was used to prepare the docking input files and Autodock4 was used to perform the docking calculations. The usual preparations were made for the protein, such as removing water and all the co-crystalized molecules, adding polar hydrogens, and Kollman united atom charges were used to neutralize the protein. The Lamarckian genetic algorithm was used to dock 250 conformations at the active site of the enzyme. The docked conformations were clustered and ranked according to the docking or binding free energy (ΔG). The enzyme–substrate interactions were elucidated and analysed using the Discovery studio 5.0 visualizer.

## 4. Conclusions

This research work has successfully filled the research gaps that were intended to be addressed, and the information gathered within can be very beneficial to the scientific community. Findings from the present work demonstrated the significant multifaceted biological properties that *B. gymnorhiza* possesses. Considering the link between DM and oxidative stress, it can be said that this plant part can be a good phytoantioxidants agent to hamper oxidative stress caused by diabetes and could even improve insulin sensitivity. Likewise, since macerated fruit was also the most potent inhibitor against the enzymes AChE, BChE, tyrosinase, elastase and α-amylase, this plant part can be a novel formulation consisting of a combined therapeutic effect to manage diabetes together with its related complications by improving cognitive performance and ameliorating cutaneous manifestations particularly diabetic dermopathy, xerosis and acanthosis nigricans. However, *B. gymnorhiza* is not a good weight-loss candidate since most extracts were inactive against pancreatic lipase enzyme. Considering the traditional usage of this mangrove plant in Mauritius, the results collected herein offer only a partial support to the traditional use since the root decoction displayed moderate α-glucosidase and relatively low α-amylase activities. Therefore, it can be concluded that *B. gymnorhiza* is not fully effective against DM itself but instead could result in a placebo effect in people. In view of the findings presented herein and taking into account that supplementing the body with radical scavengers could be a major step in impairing the progression of DM, we propose that *B. gymnorhiza* might be a promising candidate to develop new phytoagents by providing potent antioxidant compounds to fight against oxidative stress. Furthermore, the mangrove plant can open new avenues in drug discovery and designing novel pharmacophores against chronic pathological cases associated with DM.

## Figures and Tables

**Table 1:** Extraction yield (%) and total bioactive components of *B. gymnorhiza* extracts.

	Yield (%)	Total Phenolic Acid (mg CAE/g)	Total Phenolic Content (mg GAE/g)	Total Flavonoid Content (mg RE/g)	Total Flavanol (mg CE/g)	Total Tannin (mg CE/g)	Total Triterpenoids (mg OAE/g)
BLD	18.70	0.71 ± 0.04 ^g^	23.57 ± 0.03 ^f^	1.73 ± 0.03 ^d^	1.23 ± 0.02 ^e^	11.04 ± 0.07 ^d^	2.41 ± 0.51 ^f g^
BRD	7.38	14.69 ± 0.39 ^a^	177.09 ± 0.91 ^b^	0.47 ± 0.06 ^e^	13.38 ± 0.14 ^a^	90.49 ± 5.32 ^a^	29.24 ± 0.54 ^b^
BTD	7.78	5.09 ± 0.20 ^d^	84.28 ± 1.44 ^c^	0.64 ± 0.09 ^e^	2.93 ± 0.02 ^d^	35.92 ± 3.25 ^c^	9.77 ± 0.80 ^e^
BFD	8.24	3.46 ± 0.13 ^e^	84.40 ± 0.36 ^c^	5.26 ± 0.15 ^a^	3.99 ± 0.01 ^c^	45.00 ± 1.31 ^b^	12.87 ± 0.49 ^d^
BLA	11.04	0.92 ± 0.02 ^g^	47.64 ± 0.07 ^e^	1.41 ± 0.27 ^d^	1.28 ± 0.03 ^e^	14.00 ± 0.22 ^d^	1.41 ± 0.06 ^g^
BRA	4.28	10.29 ± 0.18 ^b^	184.18 ± 0.49 ^a^	2.19 ± 0.13 ^c^	13.32 ± 0.12 ^b^	98.01 ± 1.94 ^a^	23.27 ± 0.18 ^c^
BTA	4.26	2.15 ± 0.13 ^f^	77.78 ± 1.02 ^d^	0.39 ± 0.08 ^e^	3.09 ± 0.02 ^d^	17.66 ± 0.40 ^d^	4.08 ± 0.20 ^f^
BFA	11.12	9.23 ± 0.58 ^c^	185.33 ± 1.03 ^a^	3.12 ± 0.07 ^b^	13.84 ± 0.16 ^a^	97.85 ± 4.41 ^a^	36.84 ± 2.30 ^a^

Different letters (^a–g^) indicate significant differences in the tested extracts (*p* < 0.05). Values are expressed as mean ± S.D. of three parallel measurements. Abbreviations: BLD: *Bruguiera* leaf decoction; BRD: *Bruguiera* root decoction; BTD: *Bruguiera* twig decoction; BFD: *Bruguiera* fruit decoction; BLA: *Bruguiera* leaf aqueous; BRA: *Bruguiera* root aqueous; BTA: *Bruguiera* twig aqueous; BFA: *Bruguiera* fruit aqueous; GAE: Gallic acid equivalent; RE: Rutin equivalent; CE: Caffeic acid equivalent; CAE: Catechin equivalent; OAE: Oleanolic acid equivalent.

**Table 2:** Chemical composition of fruit extracts.

Compound Name	Retention Time	Decoction	Aqueous
Quinic acid	1.21	+	+
Citric acid	1.56	+	+
Brugierol	1.64	+	+
Gallocatechin (Casuarin, Gallocatechol)	4.58	+	+
Protocatechuic acid (3,4-Dihydroxybenzoic acid)	4.65	+	+
Neochlorogenic acid (5-O-Caffeoylquinic acid)	8.64	+	+
Procyanidin B isomer 1	12.05	+	+
Procyanidin B isomer 2	12.05	−	−
3-O-(4-Coumaroyl) quinic acid	12.45	+	+
Catechin	13.32	+	+
Epigallocatechin (Epigallocatechol)	13.62	+	+
Chlorogenic acid (3-O-Caffeoylquinic acid)	14.23	+	−
3-O-Feruloylquinic acid	14.50	+	+
Ampelopsin (Ampeloptin, Dihydromyricetin)	14.71	+	+
Procyanidin B isomer 3	15.51	−	−
Vanillin	15.57	+	−
Chryptochlorogenic acid (4-O-Caffeoylquinic acid)	17.08	+	+
Epicatechin	17.55	+	+
4-O-(4-Coumaroyl) quinic acid	17.75	+	−
3-(Benzoyloxy)-2-hydroxypropylglucuronic acid	18.24	−	+
4-Coumaric acid	18.52	+	+
Antiarol (3,4,5-Trimethoxyphenol)	12.45	−	+
Loliolide or Isololiolide	13.32	+	+
4-O-Feruloylquinic acid	13.62	+	+
Riboflavin	18.63	−	+
Indole-3-lactic acid	18.78	−	+
Ferulic acid	19.28	+	+
Loliolide or Isololiolide	19.31	+	+
4-Hydroxy-3-methoxycinnamaldehyde (Coniferyl aldehyde)	19.50	−	−
Sinapic acid (Sinapinic acid)	19.86	+	+
Myricetin-3-O-rutinoside	21.04	+	+
Cinchonain I isomer 1	22.28	−	−
Theaflavin	21.65	−	+
Dihydrokaempferol (Aromadendrin, Katuranin)	21.88	−	+
Cinchonain I isomer 2	22.29	−	+
Methoxy-tetrahydroxy(iso)flavone isomer 1	22.68	−	−
Isoquercitrin (Hirsutrin, Quercetin-3-O-glucoside)	22.93	−	−
Rutin (Quercetin-3-O-rutinoside)	23.01	+	+
Myricetin (Cannabisetin, Myricetol, 3,3′,4′,5,5′,7-hexahydroflavone)	24.19	−	+
Azelaic acid	24.47	+	+
Methoxy-trihydroxy(iso)flavone	24.84	−	−
Kaempferol-3-O-rutinoside (Nicotiflorin)	25.01	+	+
Gramrione (5,5′-Dimethoxy-3′,4′,7-trihydroxyflavone)	26.89	+	+
Dihydroxy-trimethoxy(iso)flavone	26.98	−	+
Quercetin (3,3′,4′,5,7-Penthahyroxyflavone)	27.19	−	+
Naringenin (4′,5,7-Trihydroxyflavanone)	27.19	+	+
Sebacic acid	27.96	−	+
Methoxy-tetrahydroxy(iso)flavone isomer 2	30.85	−	−
Bruguierol A	36.05	+	+
Lupeol caffeate	52.73	−	−
Lupeol coumarate	54.53	−	−

+: Present; −: Not present.

**Table 3:** Chemical composition of leaf extracts.

Compound Name	Retention Time	Decoction	Aqueous
Quinic acid	1.24	+	−
Brugierol	1.67	+	+
Gallocatechin	4.54	+	−
Protocatechuic acid (3,4-Dihydroxybenzoic acid)	4.71	+	+
Catechol	5.12	−	+
Genistic acid (2,5-Dihydroxybenzoic acid)	8.97	−	+
Neochlorogenic acid (5-O-Caffeoylquinic acid)	8.66	+	−
Procyanidin B isomer 1	12.07	+	−
3-O-(4-Coumaroyl) quinic acid	12.46	+	−
Catechin	13.31	+	−
Epigallocatechin	13.62	+	−
Chlorogenic acid (3-O-Caffeoylquinic acid)	14.24	+	−
Dihydroxybenzoic acid isomer	14.34	−	+
Caffeic acid	14.48	+	+
3-O-Feruloylquinic acid	15.22	+	+
Procyanidin B	15.49	+	−
Vanillin	15.57	+	−
Chryptochlorogenic acid (4-O-Caffeoylquinic acid)	17.08	+	+
Epicatechin	17.52	+	−
4-O-(4-Coumaroyl) quinic acid	17.60	+	−
3-(Benzoyloxy)-2-hydroxypropylglucuronic acid	14.34	+	−
4-Coumaric acid	17.73	+	−
Antiarol (3,4,5-Trimethoxyphenol)	17.92	+	+
Loliolide or Isololiolide	18.23	+	+
4-O-Feruloylquinic acid	18.49	+	+
Riboflavin	18.64	+	−
Cinchonain I isomer 1	18.90	+	−
Ferulic acid	19.26	+	−
Taxifolin (Didydroquercetin)	19.29	−	−
Loliolide or Isololiolide	19.50	+	+
Dimethoxy-trihydroxy(iso)flavone-O-hexoside isomer 1	20.14	+	−
Dimethoxy-trihydroxy(iso)flavone-O-hexoside isomer	20.72	+	−
Isoferulic acid	20.30	−	−
Dihydroxy-methoxy(iso)flavone-O-hexoside	20.72	−	+
Quercetin-O-dirhamnosylhexoside	20.81	+	−
Myricetin-3-O-rutinoside	21.03	+	−
Cinchonain I isomer 2	21.30	+	−
Kaempferol-O-dirhamnosylhexoside	22.11	+	−
Cinchonain I isomer 3	22.30	+	−
Methoxy-tetrahydroxy(iso)flavone isomer 1	22.67	+	+
Isoquercitrin (Hirsutrin, Quercetin-3-O-glucoside)	22.93	+	−
Dimethoxy-trihydroxy(iso)flavone-O-hexoside isomer 2	23.01	+	−
Rutin (Quercetin-3-O-rutinoside)	23.05	+	−
Apigenin-O-rhamnosylhexoside	24.48	+	−
Azelaic acid	24.48	+	+
Methoxy-trihydroxy(iso)flavone	24.70	+	+
Kaempferol-3-O-rutinoside (Nicotiflorin)	24.85	+	−
Cinchonain I isomer 4	24.95	+	−
Gramrione (5,5′-Dimethoxy-3′,4′,7-trihydroxyflavone)	25.03	+	+
Dimethoxy-trihydroxy(iso)flavone-O-hexoside isomer 3	26.00	+	−
Dihydroxy-methoxy(iso)flavone	26.51	+	+
Dihydroxy-dimethoxy(iso)flavone	26.85	+	+
Dihydroxy-trimethoxy(iso)flavone	26.89	+	+
Quercetin (3,3′, 4′, 5, 7-Pentahydroxylflavone)	28.08	−	−
Naringenin (4′,5,7-Trihydroxyflavanone)	29.16	−	−
Luteolin (3′,4′,5,7-Tetrahydroxyflavone)	20.72	−	+
Methoxy-tetrahydroxy(iso)flavone isomer 2	20.81	+	+
Dimethoxy-trihydroxy(iso)flavone-O-rhamnoside	21.03	+	−
Kaempferol (3,4′,5,7-Tetrahydroxyflavone)	29.30	−	−
Apigenin (4′,5,7-Trihydroxyflavone)	29.66	−	+
Tricin (3′,5′-Dimethoxy-4′,5,7-trihydroxyflavone)	29.84	−	−
Bruguierol A	36.06	+	+

+: Present; −: Not present.

**Table 4:** Chemical composition of root extracts.

Compound Name	Retention Time	Decoction	Aqueous
Quinic acid	1.27	+	+
Citric acid	1.58	+	+
Brugierol	1.64	+	+
Gallocatechin	4.55	+	+
Unidentified compound	4.64	+	+
Protocatechuic acid (3,4-Dihydroxybenzoic acid)	4.67	+	+
Neochlorogenic acid (5-O-Caffeoylquinic acid)	8.59	+	+
Syringic acid-O-hexoside isomer 1	10.62	+	+
Prodelphinidin C	10.83	+	+
Unidentified compound	13.12	−	+
Catechin	13.30	+	+
Epigallocatechin	13.61	+	+
Chlorogenic acid (3-O-Caffeoylquinic acid)	14.22	+	+
Caffeic acid	14.33	−	+
Unidentified compound	15.00	−	+
Procyanidin B isomer 1	16.96	+	+
Vanillin	15.54	+	−
Chryptochlorogenic acid (4-O-Caffeoylquinic acid)	15.56	+	+
Syringic acid	15.81	−	−
Procyanidin C	16.96	+	+
Epicatechin	17.07	+	+
4-Coumaric acid	17.71	−	−
3-(Benzoyloxy)-2-hydroxypropylglucuronic acid	17.59	+	+
Antiarol (3,4,5-Trimethoxyphenol)	17.91	+	+
3,4-Dihydro-3-hydroxy-7-methoxy-2H-1,5-benzodithiepine-6,9-dione	18.23	−	+
Cinchonain I isomer 1	18.90	+	+
Epiafzelechin	19.04	+	+
Ferulic acid	19.27	+	+
Taxifolin (Dihydroquercetin)	17.91	−	+
Isoferulic acid	20.32	−	−
4-Hydroxy-3-methoxycinnamaldehyde (Coniferyl aldehyde)	20.02	+	−
Procyanidin B isomer 2	20.56	−	−
Trihydroxystilbene	20.86	−	−
Myricetin-3-O-rutinoside	21.03	+	+
Cinchonain I isomer 2	21.07	+	+
Cinchonain I isomer 3	22.30	+	+
Rutin (Quercetin-3-O-rutinoside)	23.01	+	+
Azelaic acid	24.47	+	+
Methoxy-trihydroxy(iso)flavone	24.73	+	−
Kaempferol-3-O-rutinoside (Nicotiflorin)	24.85	−	−
Cinchonain I isomer 4	24.95	+	+
Gramrione (5,5′-Dimethoxy-3′,4′,7-trihydroxyflavone)	25.04	+	−
Quercetin (3,3′,4′,5,7-Pentahydroxyflavone)	26.97	−	−
Naringenin (4′,5,7-Trihydroxyflavanone)	27.19	−	−
3-O-Methylellagic acid-4′-O-rhamnoside	25.33	−	−
Sebacic acid	28.00	+	+
Phloretin	28.14	−	+
Tricin (3′,5′-Dimethoxy-4′,5,7-trihydroxyflavone)	29.84	−	−
Norstictic acid	32.46	−	+
Methyl-trihydroxyxanthone	32.88	−	−
16,17-Dihydroxy-9(11)-kauren-19-al or Steviol	33.77	+	+
1-Hydroxy-8(14)-isopimaren-1,15,16-triol or isomer	33.86	+	+
13-Hydroxy-16-kauren-19-al or isomer	35.50	+	+
Bruguierol A	36.07	+	+
1-Hydroxy-8(14)-isopimaren-1,15,16-triol or isomer	36.39	+	+
Dihydroxy-methoxy-methylxanthone	37.84	−	+
1-Hydroxy-8(14)-isopimaren-1,15,16-triol or isomer	38.07	+	+
13-Hydroxy-16-kauren-19-al or isomer	39.38	+	+
Unidentified xanthone	43.29	+	+
Isopimar-7-en-15,16-diol or isomer	43.71	+	+

+: Present; −: Not present.

**Table 5:** Chemical composition of twig extracts.

Compound Name	Retention Time	Decoction	Aqueous
Quinic acid	1.19	+	−
Citric acid	1.55	+	−
Brugierol	1.66	+	+
Gallocatechin	4.58	+	−
Protocatechuic acid (3,4-Dihydroxybenzoic acid)	4.70	+	+
Neochlorogenic acid (5-O-Caffeoylquinic acid)	8.69	+	−
Syringic acid-O-hexoside isomer 1	9.93	+	−
Syringic acid-O-hexoside isomer 2	10.66	−	−
Catechin	13.33	+	−
Epigallocatechin	13.64	+	−
Chlorogenic acid (3-O-Caffeoylquinic acid)	14.21	+	−
Caffeic acid	14.32	+	+
Vanillin	15.19	−	−
Procyanidin B	15.21	+	−
Chryptochlorogenic acid (4-O-Caffeoylquinic acid)	15.56	+	−
Syringic acid	15.81	+	−
Ehyl syringate	16.95	−	−
Procyanidin C	17.07	+	−
Epicatechin	17.59	+	−
3-(Benzoyloxy)-2-hydroxypropylglucuronic acid	17.75	+	−
4-Coumaric acid	17.93	+	−
Antiarol (3,4,5-Trimethoxyphenol)	18.25	+	+
3,4-Dihydro-3-hydroxy-7-methoxy-2H-1,5-benzodithiepine-6,9-dione	18.64	+	−
Riboflavin	18.90	−	+
Cinchonain I isomer 1	19.29	+	−
Ferulic acid	19.50	+	−
Loliolide or Isololiolide	15.56	+	−
Coumarin	19.73	−	−
4-Hydroxy-3-methoxycinnamaldehyde (Coniferyl aldehyde)	19.99	−	−
3,5-Dimethoxy-4-hydroxycinnamaldehyde (Sinapyl aldehyde)	20.44	−	−
Isoquercitrin (Hirsutin, Quercetin-3-O-glucoside)	22.94	−	−
Cinchonain I isomer 2	24.95	+	−
Cinchonain I isomer 3	22.29	−	−
Rutin (Quercetin-3-O-rutinoside)	23.04	+	−
Azelaic acid	24.48	+	+
Methoxy-trihydroxy(iso)flavone	24.72	+	−
Kaempferol-3-O-rutinoside (Nicotiflorin)	24.86	+	−
Cinchonain I isomer 4	24.95	−	−
Gramrione (5,5′-Dimethoxy-3′,4′,7-trihydroxyflavone)	25.04	+	−
Dihydroxy-methoxy(iso)flavone	26.51	+	−
Dihydroxy-dimethoxy(iso)flavone	26.85	−	−
Dihydroxy-trimethoxy(iso)flavone	26.91	−	−
Naringenin (4′,5,7-Trihydroxyflavanone)	27.20	−	−
16,17-Dihydroxy-9(11)-kauran-19-al	32.40	+	+
16,17-Dihydroxy-9(11)-kauren-19-al or Steviol	33.78	+	+
1-Hydroxy-8(14)-isopimaren-1,15,16-triol or isomer	34.79	−	−
Methyl 16α,17-dihydroxy-9(11)-kauren-19-oate or isomer	35.51	+	−
13-Hydroxy-16-kauren-19-al or isomer	35.67	+	−
16,17-Dihydroxy-9(11)-kauran-19-al isomer	36.08	+	−
Bruguierol A	36.14	+	+
Methyl 16α,17-dihydroxy-9(11)-kauren-19-oate or isomer	34.79	+	−
1-Hydroxy-8(14)-isopimaren-1,15,16-triol or isomer	36.38	−	−
13-Hydroxy-16-kauren-19-al or isomer	38.06	−	−
Isopimar-7-en-15,16-diol or isomer	38.31	−	−
Lupeol caffeate	52.71	−	−
Lupeol coumarate	54.51	−	−

+: Present; −: Not present.

**Table 6:** Antioxidant properties of *B. gymnorhiza* extracts.

	DPPH (mg TE/g)	ABTS (mg TE/g)	CUPRAC (mg TE/g)	FRAP (mg TE/g)	Phosphomolybdenum (mmol TE/g)	Chelating Activity (mg EDTAE/g)
BLD	28.30 ± 0.30 ^f^	28.82 ± 1.28 ^e^	67.14 ± 0.17 ^g^	46.08 ± 1.06 ^h^	0.40 ± 0.01 ^e^	23.69 ± 0.40 ^b^
BRD	451.72 ± 3.71 ^c^	448.09 ± 18.47 ^a^	877.30 ± 13.82 ^b^	547.22 ± 7.59 ^b^	3.70 ± 0.08 ^b^	7.35 ± 1.06 ^d^
BTD	90.34 ± 1.05 ^d^	123.16 ± 3.90 ^b^	246.52 ± 6.65 ^d e^	167.10 ± 1.03 ^e^	1.67 ± 0.03 ^c^	21.90 ± 1.08 ^b^
BFD	84.01 ± 3.35 ^d^	113.79 ± 5.58 ^b c^	271.14 ± 7.96 ^d^	185.60 ± 5.35 ^d^	1.89 ± 0.06 ^c^	10.41 ± 1.86 ^c d^
BLA	47.93 ± 2.07 ^e f^	58.95 ± 1.01 ^d e^	116.57 ± 1.67 ^f^	79.43 ± 0.33 ^g^	1.22 ± 0.05 ^d^	12.57 ± 2.60 ^c^
BRA	472.62 ± 14.58 ^b^	437.34 ± 19.60 ^a^	814.82 ± 33.09 ^c^	497.53 ± 8.49 ^c^	3.57 ± 0.04 ^b^	14.47 ± 0.18 ^c^
BTA	56.12 ± 0.76 ^e^	87.40 ± 2.40 ^c d^	211.26 ± 6.44 ^e^	105.33 ± 0.72 ^f^	1.66 ± 0.05 ^c^	31.45 ± 1.95 ^a^
BFA	547.75 ± 10.99 ^a^	439.59 ± 19.13 ^a^	956.04 ± 11.90 ^a^	577.26 ± 4.55 ^a^	5.34 ± 0.24 ^a^	14.02 ± 2.27 ^c^

Different letters (^a–h^) indicate significant differences in the tested extracts (*p* < 0.05). Values are expressed as mean ± S.D. of three parallel measurements. Abbreviations: BLD: *Bruguiera* leaf decoction; BRD: *Bruguiera* root decoction; BTD: *Bruguiera* twig decoction; BFD: *Bruguiera* fruit decoction; BLA: *Bruguiera* leaf aqueous; BRA: *Bruguiera* root aqueous; BTA: *Bruguiera* twig aqueous; BFA: *Bruguiera* fruit aqueous; TE: Trolox equivalent; EDTAE: EDTA equivalent.

**Table 7:** Enzyme inhibitory effects of *B. gymnorhiza* extracts.

	AChE Inhibition (mg GALAE/g)	BChE Inhibition (mg GALAE/g)	Tyrosinase Inhibition (mg KAE/g)	Amylase Inhibition (mmol ACAE/g)	Glucosidase Inhibition (mmol ACAE/g)	Elastase Inhibition (mg CAE/g)	Lipase Inhibition (mg OE/g)
BLD	na	0.30 ± 0.04 ^b^	22.39 ± 3.17 ^f^	0.09 ± 0.01 ^f^	na	0.56 ± 0.08 ^c d^	na
BRD	2.56 ± 0.46 ^b^	0.57 ± 0.05 ^b^	74.16 ± 3.70 ^c^	0.10 ± 0.01 ^e f^	30.87 ± 0.27 ^a b^	1.85 ± 0.26 ^b^	na
BTD	1.17 ± 0.18 ^c^	0.72 ± 0.11 ^b^	31.06 ± 1.95 ^e^	0.09 ± 0.01 ^e f^	28.40 ± 2.55 ^b^	0.35 ± 0.05 ^d^	13.81 ± 5.13 ^a^
BFD	3.90 ± 0.14 ^a^	2.85 ± 0.74 ^a^	101.93 ± 2.45 ^b^	0.70 ± 0.01 ^b^	na	2.76 ± 0.52 ^a^	na
BLA	na	na	58.72 ± 3.21 ^d^	0.10 ± 0.01 ^e^	1.41 ± 0.14 ^d^	na	na
BRA	2.13 ± 0.34 ^b^	0.32 ± 0.05 ^b^	60.74 ± 1.73 ^d^	0.13 ± 0.01 ^d^	31.18 ± 0.06 ^a^	0.39 ± 0.02 ^d^	na
BTA	na	na	11.77 ± 1.36 ^g^	0.21 ± 0.01 ^c^	24.25 ± 0.56 ^c^	1.04 ± 0.10 ^c^	na
BFA	3.75 ± 0.03 ^a^	2.19 ± 0.13 ^a^	147.01 ± 0.78 ^a^	1.22 ± 0.01 ^a^	na	3.14 ± 0.08 ^a^	na

Different letters (^a–^^g^) indicate significant differences in the tested extracts (*p* < 0.05). Values are expressed as mean ± S.D. of three parallel measurements. Abbreviations: BLD: *Bruguiera* leaf decoction; BRD: *Bruguiera* root decoction; BTD: *Bruguiera* twig decoction; BFD: *Bruguiera* fruit decoction; BLA: *Bruguiera* leaf aqueous; BRA: *Bruguiera* root aqueous; BTA: *Bruguiera* twig aqueous; BFA: *Bruguiera* fruit aqueous; GALAE: Galantamine equivalent; KAE: Kojic acid equivalent; ACAE: Acarbose equivalent; OE: Orlistat equivalent; CAE: Catechin equivalent; na: not active.

**Table 8:** Selected compounds and their corresponding enzymes for docking calculations.

Enzyme	Compound	Chemical Structure
Tyrosinase	Brugierol	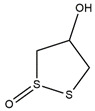
	Theaflavin	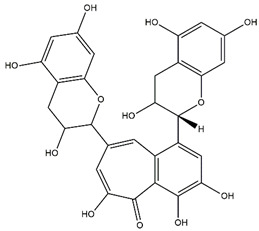
	Bruguierol A	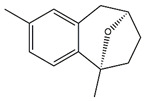
	Sebacic acid	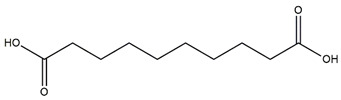
AchE	Sinapic acid	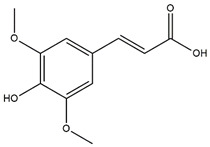
BchE	Sinapic acid	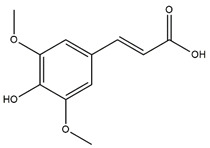
Elastase	Sebacic acid	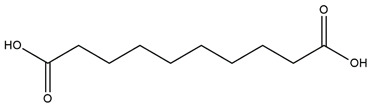

**Table 9:** Binding free energy and calculated inhibition constant of the docked compounds.

Enzyme	Compound	Binding Free Energy (kcal/mol)	Inhibition Constant (K_i_)
Tyrosinase	Brugierol	−2.34	19.23 mM
	Theaflavin	−6.59	14.86 µM
	Bruguierol A	−6.70	12.26 µM
	Sebacic acid	−5.26	139.04 µM
AchE	Sinapic acid	−6.74	11.56 µM
BchE	Sinapic acid	−6.36	21.80 µM
Elastase	Sebacic acid	−4.41	581.30 µM

## Data Availability

Not applicable.

## Supplementary Materials

The following supporting information can be downloaded at: https://www.mdpi.com/article/10.3390/molecules27062000/s1. Tables S1–S8 show all details for identified compounds. Figures S1–S16 show all chromatograms of UHPLC-ESI-MS/MS of all extracts.

## Sample Availability

Samples of the compounds are not available from the authors.
