# Supplementary material for: Towards the Pharmacological Validation and Phytochemical Profiling of the Decoction and Maceration of Bruguiera gymnorhiza (L.) Lam.—A Traditionally Used Medicinal Halophyte
Source: Molecules. 2022 Mar 20;27(6):2000. doi: 10.3390/molecules27062000 (PMC8951081; doi:10.3390/molecules27062000)
Supplement: Supplementary file 1 [file molecules-27-02000-s001.zip › molecules-1626415-Supplementary.pdf]

Table S1. Chemical composition of fruit decoction.

No.	Name	Formula	Rt	$[M + H]^+$	$[M - H]^-$	Fragment 1	Fragment 2	Fragment 3	Fragment 4	Fragment 5
1	Quinic acid	C7H12O6	1,21		191,05557	173,0446	127,0389	111,0437	93,0332	85,0280
2	Citric acid	C6H8O7	1,56		191,01918	173,0084	129,0183	111,0075	87,0073	85,0281
3	Brugierol	C3H6O2S2	1,64	138,98875		120,9779	102,9675	92,9832	86,9904	
4	Gallocatechin (Casuarin, Gallocatechol)	C15H14O7	4,58		305,06613	261,0769	219,0661	179,0343	137,0234	125,0232
5	Protocatechuic acid (3,4-Dihydroxybenzoic acid)	C7H6O4	4,65		153,01879	109,0282	108,0204	91,0177	81,0329	
6	Neochlorogenic acid (5-O-Caffeoylquinic acid)	C16H18O9	8,64	355,10291		163,0390	145,0286	135,0443	117,0338	89,0390
7	Procyanidin B	C30H26O12	12,05		577,13460	425,0889	407,0782	289,0726	161,0236	125,0231
8	3-O-(4-Coumaroyl)quinic acid	C16H18O8	12,45		337,09235	191,0556	173,0448	163,0391	119,0489	93,0331
9[1]	Catechin	C15H14O6	13,32		289,07121	245,0820	205,0504	203,0709	125,0232	109,0282
10[1]	Epigallocatechin (Epigallocatechol)	C15H14O7	13,62		305,06613	261,0769	219,0659	179,0342	137,0234	125,0232
11[1]	Chlorogenic acid (3-O-Caffeoylquinic acid)	C16H18O9	14,23	355,10291		163,0390	145,0286	135,0443	117,0338	89,0389
12	3-O-Feruloylquinic acid	C17H20O9	14,50		367,10291	193,0503	191,0560	173,0440	134,0363	93,0330
13	Ampelopsin (Ampeloptin, Dihydromyricetin)	C15H12O8	14,71		319,04540	301,0364	193,0138	178,9974	153,0182	125,0232
14[1]	Vanillin	C8H8O3	15,51	153,05517		125,0600	111,0445	110,0365	93,0340	65,0393

15	Chryptochlorogenic acid (4-O-Caffeoylquinic acid)	$C_{16}H_{18}O_9$	15,57	355,10291		163,0391	145,0286	135,0443	117,0338	89,0390
16[1]	Epicatechin	$C_{15}H_{14}O_6$	17,08		289,07121	245,0822	205,0501	203,0707	125,0232	109,0282
17	4-O-(4-Coumaroyl)quinic acid	$C_{16}H_{18}O_8$	17,55		337,09235	191,0558	173,0446	163,0391	119,0490	93,0331
18[1]	4-Coumaric acid	$C_9H_8O_3$	17,75		163,03952	119,0490	93,0332			
19	Loliolide or Isololiolide	$C_{11}H_{16}O_3$	18,24	197,11777		179,1068	161,0963	135,1169	133,1016	107,0860
20	4-O-Feruloylquinic acid	$C_{17}H_{20}O_9$	18,52		367,10291	193,0501	191,0567	173,0447	134,0361	93,0330
21	Ferulic acid	$C_{10}H_{10}O_4$	19,31		193,05009	178,0265	149,0596	137,0233	134,0361	121,0281
22	Loliolide or Isololiolide	$C_{11}H_{16}O_3$	19,50	197,11777		179,1068	161,0962	135,1171	133,1014	107,0859
23[1]	Sinapic acid (Sinapinic acid)	$C_{11}H_{12}O_5$	19,86	225,07630		207,0654	192,0421	175,0393	147,0442	119,0495
24	Myricetin-3-O-rutinoside	$C_{27}H_{30}O_{17}$	21,04		625,14048	317,0305	316,0229	287,0203	271,0253	178,9979
25[1]	Rutin (Quercetin-3-O-rutinoside)	$C_{27}H_{30}O_{16}$	23,03	611,16122		465,0963	303,0503	129,0554	85,0290	71,0499
26	Azelaic acid	$C_9H_{16}O_4$	24,49		187,09704	169,0866	143,1065	125,0959	123,0803	97,0646
27	Kaempferol-3-O-rutinoside (Nicotiflorin)	$C_{27}H_{30}O_{15}$	24,86		593,15065	327,0529	285,0430	284,0342	255,0303	227,0347
28	Gramrione (5,5'-Dimethoxy-3',4',7-trihydroxyflavone)	$C_{17}H_{14}O_7$	25,04		329,06613	314,0440	299,0194	271,0249		
29[1]	Naringenin (4',5,7-Trihydroxyflavanone)	$C_{15}H_{12}O_5$	27,23		271,06065	177,0186	151,0031	119,0488	107,0123	93,0333
30	Sebacic acid	$C_{10}H_{18}O_4$	28,00		201,11268	183,1023	157,1219	139,1118	111,0801	
31	Bruguierol A	$C_{12}H_{14}O_2$	36,08	191,10721		173,0968	161,0960	147,0806	135,0807	107,0496

Table S2. Chemical composition of fruit maceration.

No.	Name	Formula	Rt	$[M + H]^+$	$[M - H]^-$	Fragment 1	Fragment 2	Fragment 3	Fragment 4	Fragment 5
1	Quinic acid	C7H12O6	1,26		191,05557	173,0446	127,0388	111,0436	93,0330	85,0279
2	Citric acid	C6H8O7	1,30		191,01918	173,0082	129,0181	111,0074	87,0072	85,0280
3	Brugierol	C3H6O2S2	1,66	138,98875		120,9779	102,9675	92,9832	86,9904	
4	Gallocatechin (Casuarin, Gallocatechol)	C15H14O7	4,54		305,06613	261,0771	219,0653	179,0342	137,0232	125,0230
5	Protocatechuic acid (3,4-Dihydroxybenzoic acid)	C7H6O4	4,65		153,01879	109,0281	108,0203	91,0173	81,0331	
6	Neochlorogenic acid (5-O-Caffeoylquinic acid)	C16H18O9	8,68	355,10291		163,0390	145,0285	135,0442	117,0337	89,0390
7	Procyanidin B	C30H26O12	12,05		577,13460	425,0880	407,0774	289,0722	161,0233	125,0230
8	3-O-(4-Coumaroyl)quinic acid	C16H18O8	12,44		337,09235	191,0555	173,0444	163,0390	119,0489	93,0331
9[1]	Catechin	C15H14O6	13,30		289,07121	245,0821	205,0499	203,0708	125,0230	109,0280
10[1]	Epigallocatechin (Epigallocatechol)	C15H14O7	13,60		305,06613	261,0755	219,0658	179,0328	137,0235	125,0229
11	3-O-Feruloylquinic acid	C17H20O9	14,46		367,10291	193,0500	191,0555	173,0444	134,0361	93,0329
12	Ampelopsin (Ampeloptin, Dihydromyricetin)	C15H12O8	14,70		319,04540	301,0348	193,0135	178,9977	153,0181	125,0229
13	Chryptochlorogenic acid (4-O-Caffeoylquinic acid)	C16H18O9	15,55	355,10291		163,0389	145,0285	135,0443	117,0340	89,0390
14[1]	Epicatechin	C15H14O6	17,07		289,07121	245,0818	205,0504	203,0705	125,0231	109,0280
15	3-(Benzoyloxy)-2-hydroxypropylglucuronic acid	C16H20O10	17,59		371,09783	249,0614	231,0507	175,0235	121,0281	113,0230

16[1]	4-Coumaric acid	C9H8O3	17,74		163,03952	119,0488	93,0332			
17	Antiarol (3,4,5-Trimethoxyphenol)	C9H12O4	17,93	185,08139		170,0572	154,0624	153,0546	139,0390	125,0599
18	Loliolide or Isololiolide	C11H16O3	18,21	197,11777		179,1068	161,0964	135,1171	133,1014	107,0859
19	4-O-Feruloylquinic acid	C17H20O9	18,49		367,10291	193,0495	191,0567	173,0445	134,0362	93,0330
20	Riboflavin	C17H20N4O6	18,63	377,14611		359,1351	243,0877	200,0825	172,0871	99,0444
21	Indole-3-lactic acid	C11H11NO3	18,78		204,06607	186,0555	158,0600	142,0651	128,0494	72,9916
22	Ferulic acid	C10H10O4	19,28		193,05009	178,0261	149,0593	137,0233	134,0361	121,0281
23	Loliolide or Isololiolide	C11H16O3	19,49	197,11777		179,1068	161,0961	135,1170	133,1013	107,0859
24[1]	Sinapic acid (Sinapinic acid)	C11H12O5	19,84	225,07630		207,0653	192,0420	175,0391	147,0442	119,0494
25	Myricetin-3-O-rutinoside	C27H30O17	21,04		625,14048	317,0304	316,0226	287,0201	271,0251	178,9978
26	Theaflavin	C29H24O12	21,65		565,13461	427,1028	271,0605	259,0595	139,0391	
27	Dihydrokaempferol (Aromadendrin, Katuranin)	C15H12O6	21,88		287,05557	259,0614	243,0654	177,0547	151,0026	125,0231
28	Cinchonain I	C24H20O9	22,28		451,10291	341,0672	217,0134	189,0187		
29[1]	Rutin (Quercetin-3-O-rutinoside)	C27H30O16	23,01	611,16122		465,1014	303,0500	129,0549	85,0290	71,0498
30[1]	Myricetin (Cannabiscetin, Myricetol, 3,3',4',5,5',7-Hexahydroxyflavone)	C15H10O8	24,19		317,02974	271,0269	178,9977	151,0025	137,0232	109,0281
31	Azelaic acid	C9H16O4	24,47		187,09704	169,0860	143,1065	125,0958	123,0802	97,0645
32	Kaempferol-3-O-rutinoside (Nicotiflorin)	C27H30O15	24,84		593,15065	327,0526	285,0410	284,0331	255,0303	227,0339

33	Gramrione (5,5'-Dimethoxy-3',4',7-trihydroxyflavone)	C17H14O7	25,01		329,06613	314,0436	299,0199	271,0256		
34	Dihydroxy-trimethoxy(iso)flavone	C18H16O7	26,89		343,08178	328,0588	327,0504	313,0342	299,0560	285,0410
35[1]	Quercetin (3,3',4',5,7-Pentahydroxyflavone)	C15H10O7	26,98		301,03483	273,0414	229,0505	178,9978	151,0025	121,0281
36[1]	Naringenin (4',5,7-Trihydroxyflavanone)	C15H12O5	27,19		271,06065	177,0188	151,0027	119,0488	107,0124	93,0332
37	Sebacic acid	C10H18O4	27,96		201,11268	183,1019	157,1222	139,1117	111,0802	
38	Undecanedioic acid	C11H20O4	30,85		215,12834	197,1178	153,1273	125,0955		
39	Bruguierol A	C12H14O2	36,05	191,10721		173,0960	161,0960	147,0805	135,0806	107,0495

Table S3. Chemical composition of leaf decoction.

No.	Name	Formula	Rt	[M + H]+	[M - H]-	Fragment 1	Fragment 2	Fragment 3	Fragment 4	Fragment 5
1	Quinic acid	C7H12O6	1,24		191,05557	173,0444	127,0389	111,0439	93,0331	85,0281
2	Brugierol	C3H6O2S2	1,67	138,98875		120,9779	102,9677	92,9833	86,9905	
3	Gallocatechin	C15H14O7	4,54		305,06613	261,0768	219,0658	167,0339	137,0232	125,0232
4	Protocatechuic acid (3,4-Dihydroxybenzoic acid)	C7H6O4	4,71		153,01879	109,0282	108,0205	91,0174	81,0332	
5	Neochlorogenic acid (5-O-Caffeoylquinic acid)	C16H18O9	8,66	355,10291		163,0391	145,0286	135,0444	117,0338	89,0390
6	Procyanidin B isomer 1	C30H26O12	12,07		577,13460	425,0888	407,0739	289,0729	161,0232	125,0230
7	3-O-(4-Coumaroyl)quinic acid	C16H18O8	12,46		337,09235	191,0561	173,0456	163,0393	119,0490	93,0329

8[1]	Catechin	C15H14O6	13,31		289,07121	245,0819	205,0505	203,0712	125,0232	109,0282
9[1]	Epigallocatechin	C15H14O7	13,62		305,06613	261,0769	219,0658	167,0341	137,0235	125,0232
10[1]	Chlorogenic acid (3-O-Caffeoylquinic acid)	C16H18O9	14,24	355,10291		163,0391	145,0286	135,0443	117,0339	89,0391
11	Caffeic acid	C9H8O4	14,34		179,03444	135,0441	107,0489			
12	3-O-Feruloylquinic acid	C17H20O9	14,48		367,10291	193,0504	191,0555	173,0437	134,0363	93,0330
13	Procyanidin B isomer 2	C30H26O12	15,22		577,13460	425,0893	407,0773	289,0726	161,0232	125,0232
14[1]	Vanillin	C8H8O3	15,49	153,05517		125,0600	111,0445	110,0367	93,0341	65,0393
15	Chryptochlorogenic acid (4-O-Caffeoylquinic acid)	C16H18O9	15,57	355,10291		163,0392	145,0287	135,0444	117,0339	89,0391
16[1]	Epicatechin	C15H14O6	17,08		289,07121	245,0820	205,0502	203,0711	125,0231	109,0282
17	4-O-(4-Coumaroyl)quinic acid	C16H18O8	17,52		337,09235	191,0551	173,0447	163,0390	119,0489	93,0332
18	3-(Benzoyloxy)-2-hydroxypropylglucuronic acid	C16H20O10	17,60		371,09783	249,0617	231,0506	175,0236	121,0282	113,0231
19[1]	4-Coumaric acid	C9H8O3	17,73		163,03952	119,0490	93,0332			
20	Antiarol (3,4,5-Trimethoxyphenol)	C9H12O4	17,92	185,08139		170,0577	154,0625	153,0548	139,0393	125,0600
21	Loliolide or Isololiolide	C11H16O3	18,23	197,11777		179,1070	161,0963	135,1173	133,1014	107,0860
22	4-O-Feruloylquinic acid	C17H20O9	18,49		367,10291	193,0503	191,0567	173,0446	134,0363	93,0331
23	Riboflavin	C17H20N4O6	18,64	377,14611		359,1343	243,0876	200,0817	172,0865	99,0444
24	Cinchonain I isomer 1	C24H20O9	18,90		451,10291	341,0664	217,0143	189,0189		

25	Ferulic acid	C10H10O4	19,29		193,05009	178,0265	149,0593	137,0234	134,0364	121,0281
26	Loliolide or Isololiolide	C11H16O3	19,50	197,11777		179,1070	161,0963	135,1171	133,1015	107,0860
27	Dimethoxy-trihydroxy(iso)flavone-O-hexoside isomer 1	C23H24O12	20,14		491,11896	329,0671	328,0592	314,0446	313,0375	285,0417
28	Dihydroxy-methoxy(iso)flavone-O-hexoside	C22H22O10	20,72		445,11348	283,0620	282,0537	268,0384	240,0423	
29	Quercetin-O-dirhamnosylhexoside	C33H40O20	20,81		755,20347	300,0284	271,0244	255,0307		
30	Myricetin-3-O-rutinoside	C27H30O17	21,03		625,14048	317,0305	316,0230	287,0210	271,0254	178,9978
31	Cinchonain I isomer 2	C24H20O9	21,30		451,10291	341,0671	217,0141	189,0188		
32	Kaempferol-O-dirhamnosylhexoside	C33H40O19	22,11		739,20856	327,0514	285,0405	284,0332	255,0301	227,0348
33	Cinchonain I isomer 3	C24H20O9	22,30		451,10291	341,0652	217,0134	189,0185		
34	Methoxy-tetrahydroxy(iso)flavone isomer 1	C16H12O7	22,67		315,05048	300,0281	299,0207	272,0334	256,0378	
35[1]	Isoquercitrin (Hirsutrin, Quercetin-3-O-glucoside)	C21H20O12	22,93		463,08765	301,0356	300,0278	271,0243	255,0290	178,9979
36	Dimethoxy-trihydroxy(iso)flavone-O-hexoside isomer 2	C23H24O12	23,01		491,11896	329,0674	314,0440	313,0369	299,0203	271,0247
37[1]	Rutin (Quercetin-3-O-rutinoside)	C27H30O16	23,05	611,16122		465,1041	303,0501	129,0551	85,0290	71,0498
38	Apigenin-O-rhamnosylhexoside	C27H30O14	24,48		577,15573	269,0464	268,0389			
39	Azelaic acid	C9H16O4	24,48		187,09704	169,0863	143,1071	125,0960	123,0802	97,0646
40	Methoxy-trihydroxy(iso)flavone	C16H12O6	24,70		299,05556	284,0334	283,0255	256,0380	133,0284	
41	Kaempferol-3-O-rutinoside (Nicotiflorin)	C27H30O15	24,85		593,15065	327,0514	285,0411	284,0333	255,0302	227,0347

42	Cinchonain I isomer 4	C24H20O9	24,95		451,10291	341,0671	217,0133	189,0186		
43	Gramrione (5,5'-Dimethoxy-3',4',7-trihydroxyflavone)	C17H14O7	25,03		329,06613	314,0439	299,0205	271,0255		
44	Dimethoxy-trihydroxy(iso)flavone-O-hexoside isomer 3	C23H24O12	26,00		491,11896	329,0668	328,0600	314,0440	313,0362	285,0416
45	Dihydroxy-methoxy(iso)flavone	C16H12O5	26,51		283,06065	268,0383	267,0304	240,0427	239,0342	211,0395
46	Dihydroxy-dimethoxy(iso)flavone	C17H14O6	26,85	315,08686		300,0632	299,0554	285,0407	272,0686	257,0444
47	Dihydroxy-trimethoxy(iso)flavone	C18H16O7	26,89		343,08178	328,0595	327,0513	313,0361	299,0567	285,0411
48	Methoxy-tetrahydroxy(iso)flavone isomer 2	C16H12O7	28,08		315,05048	300,0281	272,0328	256,0368		
49	Dimethoxy-trihydroxy(iso)flavone-O-rhamnoside	C23H24O11	29,16		475,12404	328,0595	327,0515	314,0442	313,0361	285,0410
50	Bruguierol A	C12H14O2	36,06	191,10721		173,0964	161,0961	147,0808	135,0807	107,0496

Table S4. Chemical composition of leaf maceration.

No.	Name	Formula	Rt	[M + H]+	[M - H]-	Fragment 1	Fragment 2	Fragment 3	Fragment 4	Fragment 5
1	Brugierol	C3H6O2S2	1,67	138,98875		120,9778	102,9672	92,9832	86,9904	
2	Methoxy-trihydroxybenzene	C7H8O4	3,25		155,03443	140,0102	139,0024			
3	Protocatechuic acid (3,4-Dihydroxybenzoic acid)	C7H6O4	4,67		153,01879	109,0281	108,0203	91,0175	81,0332	
4	Catechol	C6H6O2	5,12		109,02896	108,0203	91,0173	81,0331		
5	Gentisic acid (2,5-Dihydroxybenzoic acid)	C7H6O4	8,97		153,01879	123,0074	109,0281	108,0203	95,0124	85,0280

6	Dihydroxybenzoic acid isomer	C7H6O4	12,48		153,01879	123,0073	109,0280	108,0202	95,0123	81,0331
7	Caffeic acid	C9H8O4	14,33		179,03444	135,0439	107,0488			
8	3-O-Feruloylquinic acid	C17H20O9	14,48		367,10291	193,0499	191,0552	173,0445	134,0361	93,0330
9	Chryptochlorogenic acid (4-O-Caffeoylquinic acid)	C16H18O9	15,59	355,10291		163,0389	145,0285	135,0441	117,0339	89,0391
10	Antiarol (3,4,5-Trimethoxyphenol)	C9H12O4	17,95	185,08139		170,0571	154,0623	153,0545	139,0390	125,0598
11	Loliolide or Isololiolide	C11H16O3	18,25	197,11777		179,1067	161,0960	135,1170	133,1014	107,0859
12	4-O-Feruloylquinic acid	C17H20O9	18,49		367,10291	193,0499	191,0567	173,0445	134,0358	93,0329
13	Loliolide or Isololiolide	C11H16O3	19,52	197,11777		179,1067	161,0962	135,1170	133,1013	107,0859
14	Isoferulic acid	C10H10O4	20,30		193,05009	178,0266	149,0598	137,0237	134,0361	
15	Methoxy-tetrahydroxy(iso)flavone isomer 1	C16H12O7	22,67		315,05048	300,0278	299,0199	272,0328	256,0371	
16	Azelaic acid	C9H16O4	24,47		187,09704	169,0856	143,1067	125,0958	123,0802	97,0645
17	Methoxy-trihydroxy(iso)flavone	C16H12O6	24,70		299,05556	284,0328	283,0251	256,0376	133,0282	
18	Gramrione (5,5'-Dimethoxy-3',4',7-trihydroxyflavone)	C17H14O7	25,01		329,06613	314,0434	299,0198	271,0249		
19	Dihydroxy-methoxy(iso)flavone	C16H12O5	26,50		283,06065	268,0379	267,0299	240,0423	239,0355	211,0393
20	Dihydroxy-dimethoxy(iso)flavone	C17H14O6	26,85	315,08686		300,0625	299,0546	285,0393	272,0674	257,0445
21	Dihydroxy-trimethoxy(iso)flavone	C18H16O7	26,88		343,08178	328,0589	327,0522	313,0357	299,0563	285,0410
22[1]	Luteolin (3',4',5,7-Tetrahydroxyflavone)	C15H10O6	27,84		285,03991	217,0500	199,0394	175,0379	151,0023	133,0280

23	Methoxy-tetrahydroxy(iso)flavone isomer 2	C16H12O7	28,07		315,05048	300,0277	272,0321	256,0368		
24[1]	Apigenin (4',5,7-Trihydroxyflavone)	C15H10O5	29,66		269,04500	225,0565	151,0026	149,0230	117,0331	107,0123
25	Bruguierol A	C12H14O2	36,05	191,10721		173,0959	161,0964	147,0799	135,0805	107,0495

Table S5. Chemical composition of root decoction.

No.	Name	Formula	Rt	$[M + H]^+$	$[M - H]^-$	Fragment 1	Fragment 2	Fragment 3	Fragment 4	Fragment 5
1	Quinic acid	C7H12O6	1,20		191,05557	173,0448	127,0390	111,0438	93,0333	85,0281
2	Citric acid	C6H8O7	1,56		191,01918	173,0087	129,0182	111,0074	87,0073	85,0280
3	Brugierol	C3H6O2S2	1,63	138,98875		120,9780	102,9677	92,9833	86,9905	
4	Gallocatechin	C15H14O7	4,37		305,06613	261,0772	219,0662	167,0340	137,0233	125,0231
5	Unidentified compound	C9H19NO6S2	4,64	302,07320		285,0464	180,0868	163,0603	145,0498	122,9936
6	Protocatechuic acid (3,4-Dihydroxybenzoic acid)	C7H6O4	4,67		153,01879	109,0282	108,0204	91,0174	81,0334	
7	Neochlorogenic acid (5-O-Caffeoylquinic acid)	C16H18O9	8,61	355,10291		163,0392	145,0287	135,0444	117,0339	89,0390
8	Syringic acid-O-hexoside	C15H20O10	10,60		359,09783	197,0451	182,0214	166,9978	153,0547	138,0312
9	Prodelphinidin C	C30H26O13	10,78		593,12952	425,0885	407,0779	289,0724	177,0185	125,0232
10[1]	Catechin	C15H14O6	13,31		289,07121	245,0822	205,0505	203,0709	125,0231	109,0282
11[1]	Epigallocatechin	C15H14O7	13,61		305,06613	261,0771	219,0661	167,0341	137,0234	125,0232

12[1]	Chlorogenic acid (3-O-Caffeoylquinic acid)	C16H18O9	14,24	355,10291		163,0392	145,0288	135,0445	117,0339	89,0393
13	Procyanidin B	C30H26O12	15,22		577,13460	425,0888	407,0778	289,0725	161,0234	125,0232
14[1]	Vanillin	C8H8O3	15,54	153,05517		125,0600	111,0445	110,0365	93,0340	65,0393
15	Chryptochlorogenic acid (4-O-Caffeoylquinic acid)	C16H18O9	15,57	355,10291		163,0392	145,0287	135,0444	117,0339	89,0390
16	Procyanidin C	C45H38O18	16,98		865,19799	577,1361	407,0777	289,0725	161,0234	125,0232
17[1]	Epicatechin	C15H14O6	17,09		289,07121	245,0821	205,0503	203,0710	125,0232	109,0282
18	3-(Benzoyloxy)-2-hydroxypropylglucuronic acid	C16H20O10	17,60		371,09783	249,0618	231,0511	175,0242	121,0283	113,0231
19	Antiarol (3,4,5-Trimethoxyphenol)	C9H12O4	17,95	185,08139		170,0575	154,0626	153,0548	139,0392	125,0600
20	Cinchonain I isomer 1	C24H20O9	18,91		451,10291	341,0672	217,0141	189,0189		
21	Epiafzelechin	C15H14O5	19,05		273,07630	255,0671	229,0860	189,0546	137,0231	97,0283
22	Ferulic acid	C10H10O4	19,29		193,05009	178,0261	149,0599	137,0227	134,0362	121,0280
23	4-Hydroxy-3-methoxycinnamaldehyde (Co-niferyl aldehyde)	C10H10O3	20,02	179,07082		161,0600	147,0443	133,0653	119,0495	55,0186
24	Myricetin-3-O-rutinoside	C27H30O17	21,03		625,14048	317,0311	316,0230	287,0207	271,0243	178,9975
25	Cinchonain I isomer 2	C24H20O9	21,27		451,10291	341,0671	217,0137	189,0188		
26	Cinchonain I isomer 3	C24H20O9	22,30		451,10291	341,0672	217,0136	189,0188		
27[1]	Rutin (Quercetin-3-O-rutinoside)	C27H30O16	23,02	611,16122		465,1030	303,0501	129,0549	85,0290	71,0498
28	Azelaic acid	C9H16O4	24,49		187,09704	169,0862	143,1069	125,0959	123,0805	97,0646

29	Methoxy-trihydroxy(iso)flavone	$C_{16}H_{12}O_6$	24,73		299,05556	284,0321	283,0264	256,0381	133,0286	
30	Cinchonain I isomer 4	$C_{24}H_{20}O_9$	24,97		451,10291	341,0673	217,0136	189,0188		
31	Gramrione (5,5'-Dimethoxy-3',4',7-trihydroxyflavone)	$C_{17}H_{14}O_7$	25,04		329,06613	314,0437	299,0195	271,0248		
32	Sebacic acid	$C_{10}H_{18}O_4$	28,00		201,11268	183,1021	157,1228	139,1118	111,0806	
33	16,17-Dihydroxy-9(11)-kauren-19-al or Steviol	$C_{20}H_{30}O_3$	33,80	319,22732		301,2166	283,2057	273,2210	255,2114	241,1951
34	1-Hydroxy-8(14)-isopimaren-1,15,16-triol or isomer	$C_{20}H_{34}O_3$	33,88	323,25862		305,2475	287,2372	269,2267	245,2269	81,0705
35	13-Hydroxy-16-kauren-19-al or isomer	$C_{20}H_{30}O_2$	35,52	303,23241		285,2216	275,2371	267,2112	257,2267	95,0862
36	Bruguierol A	$C_{12}H_{14}O_2$	36,08	191,10721		173,0963	161,0957	147,0803	135,0808	107,0497
37	1-Hydroxy-8(14)-isopimaren-1,15,16-triol or isomer	$C_{20}H_{34}O_3$	36,41	323,25862		305,2475	287,2370	269,2264	257,2264	95,0860
38	1-Hydroxy-8(14)-isopimaren-1,15,16-triol or isomer	$C_{20}H_{34}O_3$	38,10	323,25862		305,2474	287,2370	269,2267	257,2261	95,0861
39	13-Hydroxy-16-kauren-19-al or isomer	$C_{20}H_{30}O_2$	39,41	303,23241		285,2216	275,2368	267,2108	257,2266	109,1016
40	Unidentified xanthone isomer 1	$C_{18}H_{16}O_7$	41,84		343,08178	328,0592	313,0364	259,0614	231,0660	83,0124
41	Unidentified xanthone isomer 2	$C_{18}H_{16}O_7$	43,33		343,08178	328,0598	313,0364	259,0615	231,0662	83,0123
42	Isopimar-7-en-15,16-diol or isomer	$C_{20}H_{34}O_2$	43,75	307,26371		289,2531	271,2425	247,2431	231,2112	81,0705
43	Isopimar-7-en-15,16-diol or isomer	$C_{20}H_{34}O_2$	43,99	307,26371		289,2530	271,2422	247,2431	231,2105	81,0705

Table S6. Chemical composition of root maceration.

No.	Name	Formula	Rt	$[M + H]^+$	$[M - H]^-$	Fragment 1	Fragment 2	Fragment 3	Fragment 4	Fragment 5
1	Quinic acid	C7H12O6	1,27		191,05557	173,0445	127,0384	111,0437	93,0329	85,0279
2	Citric acid	C6H8O7	1,58		191,01918	173,0080	129,0181	111,0073	87,0071	85,0279
3	Brugierol	C3H6O2S2	1,64	138,99		120,9778	102,9674	92,9832	86,9904	
4	Gallocatechin	C15H14O7	4,55		305,06613	261,0760	219,0656	167,0338	137,0231	125,0230
5	Unidentified compound	C9H19NO6S2	4,64	302,0732		285,0466	180,0859	163,0602	145,0496	122,9934
6	Protocatechuic acid (3,4-Dihydroxybenzoic acid)	C7H6O4	4,67		153,01879	109,0280	108,0203	91,0173	81,0331	
7	Neochlorogenic acid (5-O-Caffeoylquinic acid)	C16H18O9	8,59	355,10291		163,0389	145,0284	135,0441	117,0334	89,0391
8	Syringic acid-O-hexoside	C15H20O10	10,6		359,10	197,0447	182,0212	166,9960	153,0544	138,0308
9	Prodelphinidin C	C30H26O13	10,8		593,13	425,0891	407,0771	289,0718	177,0182	125,0230
10	Unidentified compound	C11H14O5	13,1		225,08	207,0656	163,0753	123,0437	101,0229	
11[1]	Catechin	C15H14O6	13,3		289,07	245,0815	205,0500	203,0708	125,0230	109,0280
12[1]	Epigallocatechin	C15H14O7	13,6		305,07	261,0772	219,0657	167,0338	137,0232	125,0230
13[1]	Chlorogenic acid (3-O-Caffeoylquinic acid)	C16H18O9	14,2	355,10		163,0390	145,0282	135,0442	117,0339	89,0392
14	Caffeic acid	C9H8O4	14,3		179,03	135,0438	107,0488			
15	Unidentified compound	C11H12O4	15		207,07	163,0752	122,0359	109,0280		

16	Procyanidin B	C30H26O12	15,2		577,13	425,0884	407,0771	289,0721	161,0232	125,0230
17	Chryptochlorogenic acid (4-O-Caffeoylquinic acid)	C16H18O9	15,6	355,10		163,0389	145,0282	135,0442	117,0337	89,0389
18	Procyanidin C	C45H38O18	17		865,20	577,1346	407,0773	289,0721	161,0232	125,0230
19[1]	Epicatechin	C15H14O6	17,1		289,07	245,0815	205,0501	203,0707	125,0230	109,0280
20	3-(Benzoyloxy)-2-hydroxypropylglucuronic acid	C16H20O10	17,6		371,10	249,0613	231,0506	175,0237	121,0280	113,0229
21	Antiarol (3,4,5-Trimethoxyphenol)	C9H12O4	17,9	185,08		170,0573	154,0624	153,0546	139,0390	125,0599
22	3,4-Dihydro-3-hydroxy-7-methoxy-2H-1,5-benzodithiepine-6,9-dione	C10H10O4S2	18,2	259,01		240,9986	225,0215	208,0191	207,0111	73,0112
23	Cinchonain I isomer 1	C24H20O9	18,9		451,10	341,0667	217,0136	189,0188		
24	Epiafzelechin	C15H14O5	19		273,08	255,0652	229,0860	189,0549	137,0231	97,0281
25[1]	Taxifolin (Dihydroquercetin)	C15H12O7	19,3		303,05	285,0410	217,0512	175,0386	153,0181	125,0229
26	Ferulic acid	C10H10O4	19,3		193,05	178,0261	149,0595	137,0230	134,0361	121,0277
27	Myricetin-3-O-rutinoside	C27H30O17	21		625,14	317,0289	316,0226	287,0203	271,0246	178,9982
28	Cinchonain I isomer 2	C24H20O9	21,3		451,10	341,0673	217,0133	189,0189		
29	Cinchonain I isomer 3	C24H20O9	22,3		451,10	341,0678	217,0133	189,0184		
30[1]	Rutin (Quercetin-3-O-rutinoside)	C27H30O16	23	611,16		465,1030	303,0497	129,0546	85,0289	71,0497
31	Azelaic acid	C9H16O4	24,5		187,10	169,0856	143,1065	125,0957	123,0801	97,0644
32	Cinchonain I isomer 4	C24H20O9	25		451,10	341,0667	217,0135	189,0186		

33	Sebacic acid	C10H18O4	28		201,11	183,1018	157,1214	139,1116	111,0798	
34[1]	Phloretin	C15H14O5	28,1		273,08	229,0867	179,0340	167,0338	123,0438	119,0489
35	Norstictic acid	C18H12O9	32,5		371,04	327,0508	283,0605	255,0652	243,0291	227,0346
36	16,17-Dihydroxy-9(11)-kauren-19-al or Steviol	C20H30O3	33,8	319,23		301,2159	283,2052	273,2208	255,2103	241,1950
37	1-Hydroxy-8(14)-isopimaren-1,15,16-triol or isomer	C20H34O3	33,9	323,26		305,2472	287,2367	269,2259	245,2264	81,0704
38	13-Hydroxy-16-kauren-19-al or isomer	C20H30O2	35,5	303,23		285,2213	275,2367	267,2106	257,2259	95,0860
39	Bruguierol A	C12H14O2	36,1	191,11		173,0958	161,0955	147,0801	135,0806	107,0495
40	1-Hydroxy-8(14)-isopimaren-1,15,16-triol or isomer	C20H34O3	36,4	323,26		305,2473	287,2368	269,2261	257,2260	95,0859
41	Dihydroxy-methoxy-methylxanthone	C15H12O5	37,8		271,06	256,0375	228,0428	227,0347		
42	1-Hydroxy-8(14)-isopimaren-1,15,16-triol or isomer	C20H34O3	38,1	323,26		305,2474	287,2368	269,2262	257,2262	95,0859
43	13-Hydroxy-16-kauren-19-al or isomer	C20H30O2	39,4	303,23		285,2211	275,2376	267,2109	257,2264	109,1014
44	Unidentified xanthone	C18H16O7	43,3		343,08	328,0589	313,0362	259,0613	231,0656	83,0123
45	Isopimar-7-en-15,16-diol or isomer	C20H34O2	43,7	307,26		289,2524	271,2422	247,2413	231,2105	81,0704
46	Isopimar-7-en-15,16-diol or isomer	C20H34O2	44	307,26		289,2527	271,2421	247,2422	231,2106	81,0704

Table S7. Chemical composition of twigs decoction.

No.	Name	Formula	Rt	$[M + H]^+$	$[M - H]^-$	Fragment 1	Fragment 2	Fragment 3	Fragment 4	Fragment 5
1	Quinic acid	$C_7H_{12}O_6$	1,19		191,05557	173,0445	127,0389	111,0438	93,0331	85,0280
2	Citric acid	$C_6H_8O_7$	1,57		191,01918	173,0082	129,0182	111,0074	87,0073	85,0280
3	Brugierol	$C_3H_6O_2S_2$	1,66	138,98875		120,9780	102,9676	92,9832	86,9905	
4	Gallocatechin	$C_{15}H_{14}O_7$	4,53		305,06613	261,0772	219,0660	167,0341	137,0234	125,0232
5	Protocatechuic acid (3,4-Dihydroxybenzoic acid)	$C_7H_6O_4$	4,69		153,01879	109,0282	108,0204	91,0175	81,0333	
6	Neochlorogenic acid (5-O-Caffeoylquinic acid)	$C_{16}H_{18}O_9$	8,67	355,10291		163,0391	145,0288	135,0444	117,0339	89,0389
7	Syringic acid-O-hexoside	$C_{15}H_{20}O_{10}$	10,61		359,09783	197,0451	182,0215	166,9977	153,0547	138,0312
8	Procyanidin B isomer 1	$C_{30}H_{26}O_{12}$	12,06		577,13460	425,0896	407,0772	289,0723	161,0235	125,0232
9[1]	Catechin	$C_{15}H_{14}O_6$	13,32		289,07121	245,0821	205,0505	203,0710	125,0231	109,0282
10	Procyanidin B isomer 2	$C_{30}H_{26}O_{12}$	13,47		577,13460	425,0889	407,0785	289,0724	161,0237	125,0232
11[1]	Epigallocatechin	$C_{15}H_{14}O_7$	13,62		305,06613	261,0770	219,0660	167,0341	137,0234	125,0232
12[1]	Chlorogenic acid (3-O-Caffeoylquinic acid)	$C_{16}H_{18}O_9$	14,23	355,10291		163,0392	145,0287	135,0443	117,0338	89,0391
13	Caffeic acid	$C_9H_8O_4$	14,36		179,03444	135,0441	107,0489			
14	Procyanidin B isomer 3	$C_{30}H_{26}O_{12}$	15,23		577,13460	425,0883	407,0778	289,0724	161,0233	125,0231
15	Chryptochlorogenic acid (4-O-Caffeoylquinic acid)	$C_{16}H_{18}O_9$	15,58	355,10291		163,0392	145,0286	135,0444	117,0340	89,0392

16	Syringic acid	C9H10O5	15,83		197,04500	182,0215	166,9976	153,0547	138,0311	123,0075
17	Procyanidin C	C45H38O18	16,95		865,19799	577,1341	407,0781	289,0731	161,0235	125,0231
18[1]	Epicatechin	C15H14O6	17,09		289,07121	245,0820	205,0503	203,0711	125,0231	109,0282
19	3-(Benzoyloxy)-2-hydroxypropylglucuronic acid	C16H20O10	17,60		371,09783	249,0617	231,0508	175,0244	121,0282	113,0231
20[1]	4-Coumaric acid	C9H8O3	17,75		163,03952	119,0490	93,0331			
21	Antiarol (3,4,5-Trimethoxyphenol)	C9H12O4	17,94	185,08139		170,0576	154,0626	153,0548	139,0392	125,0600
22	3,4-Dihydro-3-hydroxy-7-methoxy-2H-1,5-benzodithiepine-6,9-dione	C10H10O4S2	18,25	259,00988		240,9990	225,0221	208,0188	207,0116	73,0113
23	Cinchonain I isomer 1	C24H20O9	18,90		451,10291	341,0671	217,0136	189,0186		
24	Ferulic acid	C10H10O4	19,29		193,05009	178,0264	149,0596	137,0233	134,0363	121,0281
25	Loliolide or Isololiolide	C11H16O3	19,52	197,11777		179,1069	161,0961	135,1172	133,1015	107,0860
26[1]	Rutin (Quercetin-3-O-rutinoside)	C27H30O16	23,04	611,16122		465,1030	303,0503	129,0551	85,0291	71,0498
27	Azelaic acid	C9H16O4	24,49		187,09704	169,0862	143,1067	125,0959	123,0800	97,0645
28	Methoxy-trihydroxy(iso)flavone	C16H12O6	24,72		299,05556	284,0337	283,0256	256,0377	133,0284	
29	Kaempferol-3-O-rutinoside (Nicotiflorin)	C27H30O15	24,86		593,15065	327,0529	285,0410	284,0339	255,0293	227,0342
30	Cinchonain I isomer 2	C24H20O9	24,95		451,10291	341,0669	217,0137	189,0189		
31	Gramrione (5,5'-Dimethoxy-3',4',7-trihydroxyflavone)	C17H14O7	25,04		329,06613	314,0440	299,0205	271,0254		
32	Dihydroxy-trimethoxy(iso)flavone	C18H16O7	26,91		343,08178	328,0586	327,0498	313,0357	299,0565	285,0412

33	16,17-Dihydroxy-9(11)-kauran-19-al	C20H32O3	32,40	321,24297		303,2312	285,2217	273,2209	267,2109	255,2106
34	16,17-Dihydroxy-9(11)-kauren-19-al or Steviol	C20H30O3	33,78	319,22732		301,2171	283,2060	273,2213	255,2105	241,1952
35	Methyl 16α,17-dihydroxy-9(11)-kauren-19-oate or isomer	C21H32O4	34,79	349,23788		331,2267	299,2008	271,2060	253,1950	121,1015
36	13-Hydroxy-16-kauren-19-al or isomer	C20H30O2	35,51	303,23241		285,2217	275,2368	267,2104	257,2266	95,0861
37	16,17-Dihydroxy-9(11)-kauran-19-al isomer	C20H32O3	35,67	321,24297		303,2325	285,2215	273,2215	267,2100	255,2114
38	Bruguierol A	C12H14O2	36,08	191,10721		173,0962	161,0965	147,0808	135,0807	107,0497
39	Methyl 16α,17-dihydroxy-9(11)-kauren-19-oate or isomer	C21H32O4	36,14	349,23788		331,2266	299,2004	271,2060	253,1950	121,1012

Table S8. Chemical composition of twigs maceration.

No.	Name	Formula	Rt	$[M + H]^+$	$[M - H]^-$	Fragment 1	Fragment 2	Fragment 3	Fragment 4	Fragment 5
1	Brugierol	C3H6O2S2	1,67	138,98875		120,9778	102,9670	92,9831	86,9903	
2	Protocatechuic acid (3,4-Dihydroxybenzoic acid)	C7H6O4	4,7		153,01879	109,0281	108,0202	91,0175	81,0331	
3	Caffeic acid	C9H8O4	14,4		179,03444	135,0439	107,0487			
4	Antiarol (3,4,5-Trimethoxyphenol)	C9H12O4	17,9	185,08139		170,0571	154,0624	153,0546	139,0391	125,0599
5	Riboflavin	C17H20N4O6	18,7	377,14611		359,1335	243,0877	200,0824	172,0867	99,0444
6	Azelaic acid	C9H16O4	24,5		187,09704	169,0860	143,1062	125,0958	123,0798	97,0643
7	16,17-Dihydroxy-9(11)-kauran-19-al	C20H32O3	32,4	321,24297		303,2312	285,2211	273,2219	267,2108	255,2104

8	16,17-Dihydroxy-9(11)-kauren-19-al or Steviol	C20H30O 3	33, 8	319,22 732		301,215 8	283,206 4	273,221 7	255,209 7	241,195 3
9	Bruguierol A	C12H14O 2	36, 1	191,10 721		173,096 3	161,095 9	147,080 5	135,080 6	107,049 5

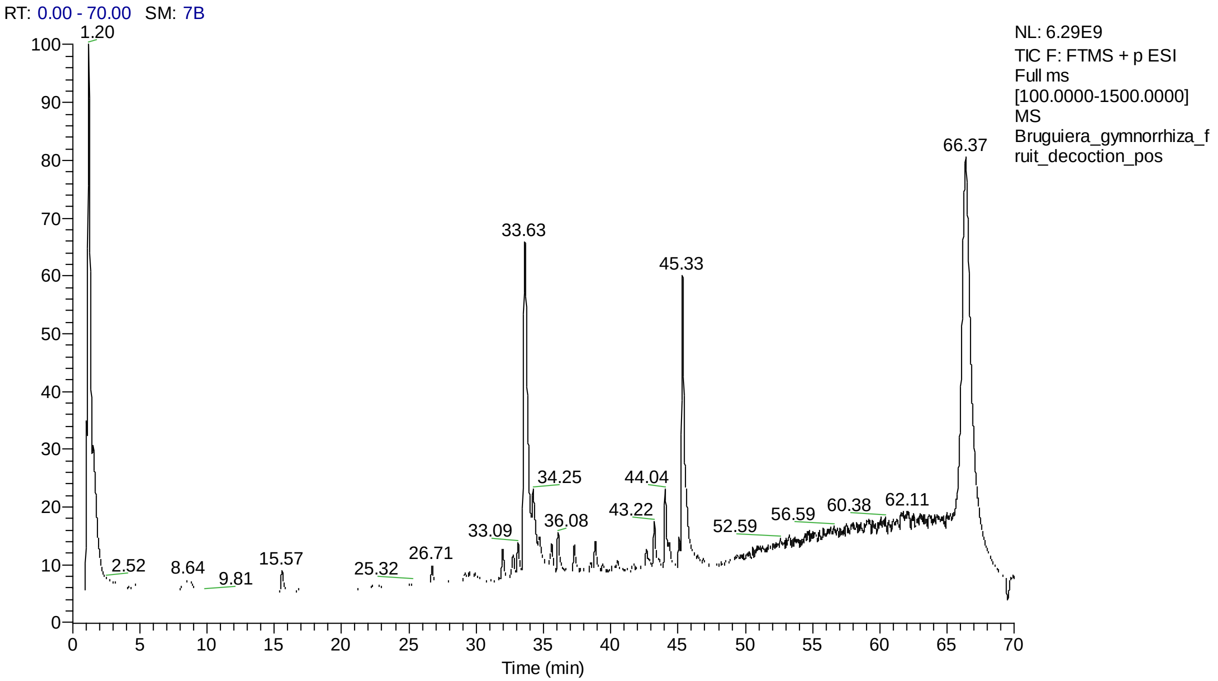

Figure S1. *Bruguiera gymnorrhiza* fruit decoction TIC positive.

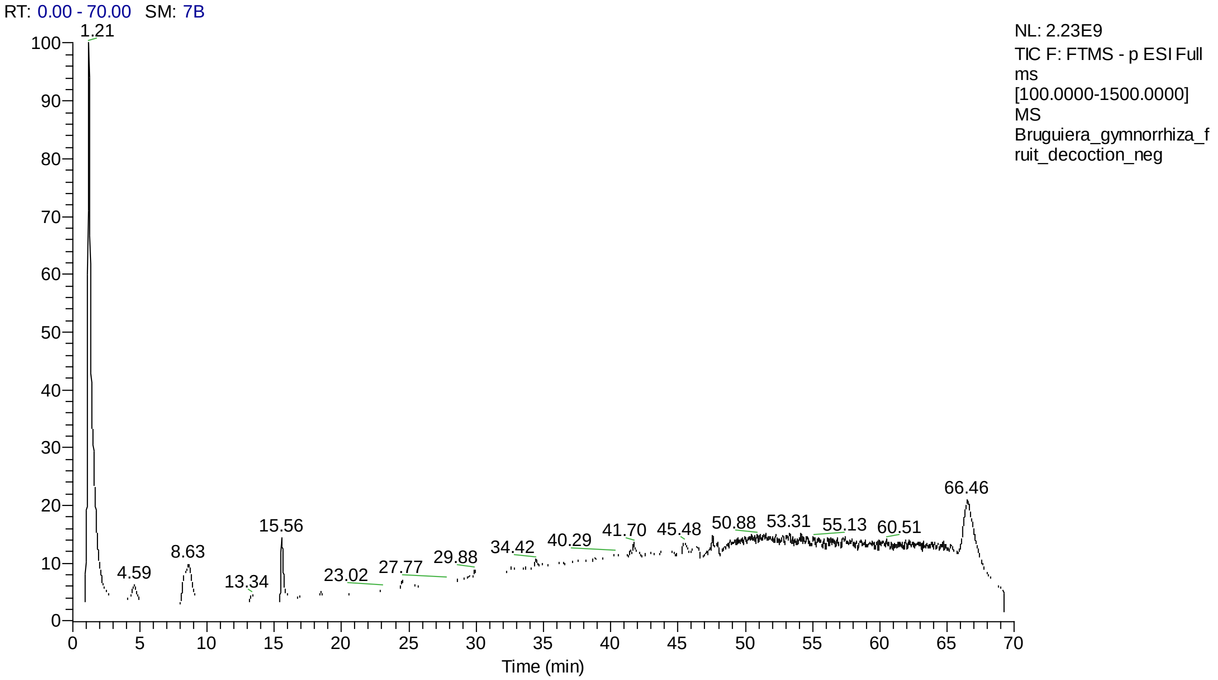

Figure S2. Bruguiera gymnorrhiza fruit decoction TIC negative.

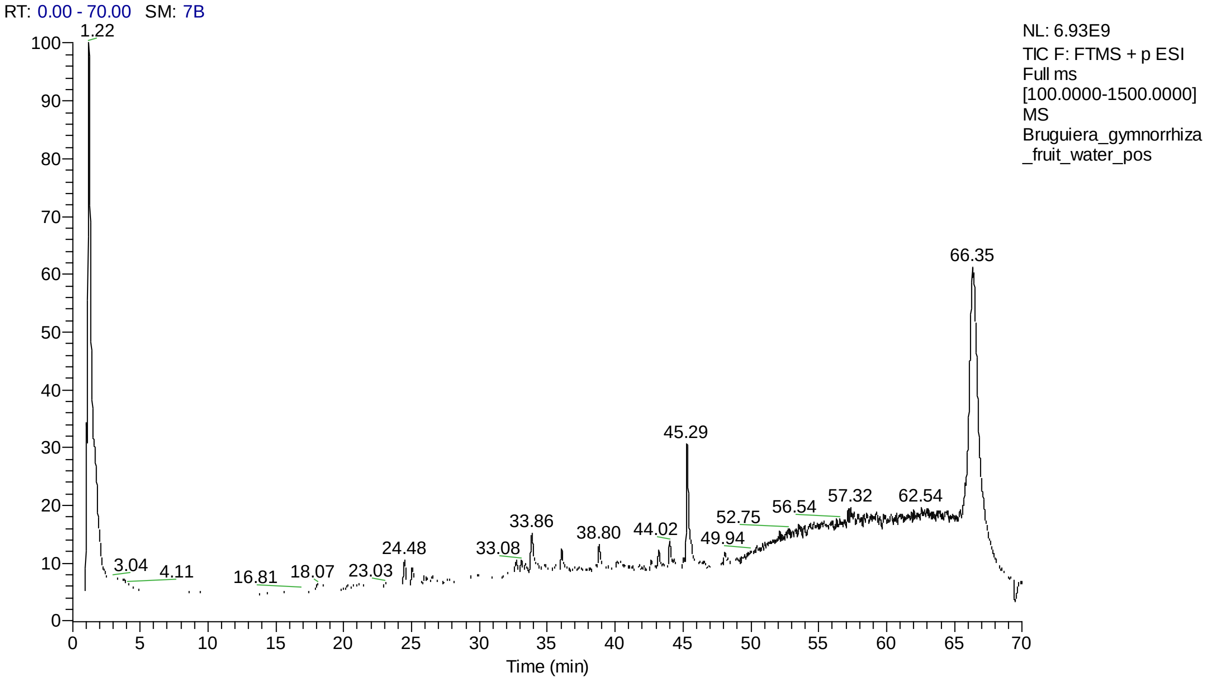

Figure S3. Bruguiera gymnorrhiza fruit water TIC positive.

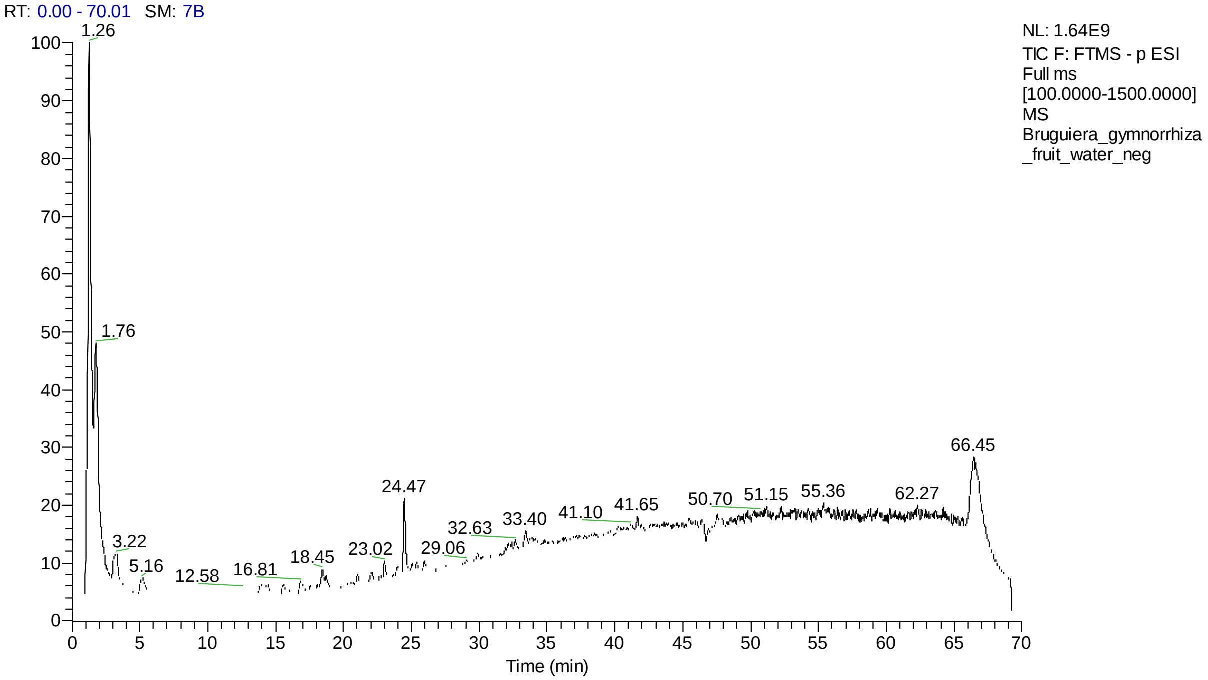

Figure S4. Bruguiera gymnorrhiza fruit water TIC negative.

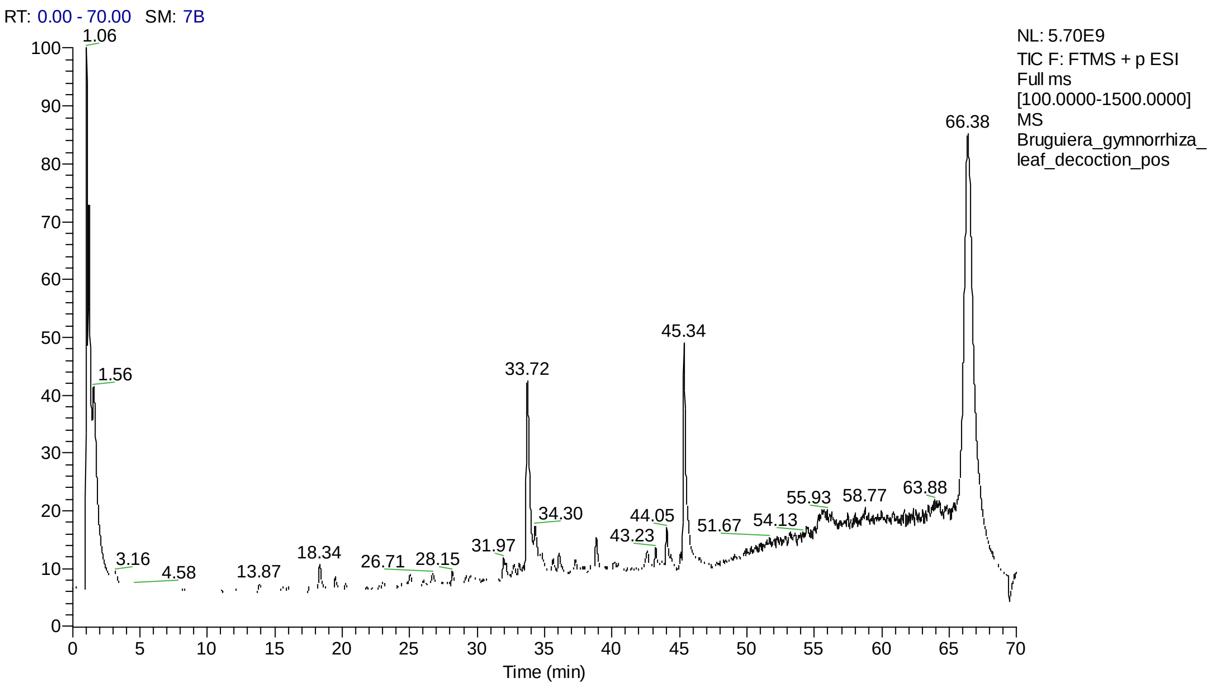

Figure S5. Bruguiera gymnorrhiza leaf decoction TIC positive.

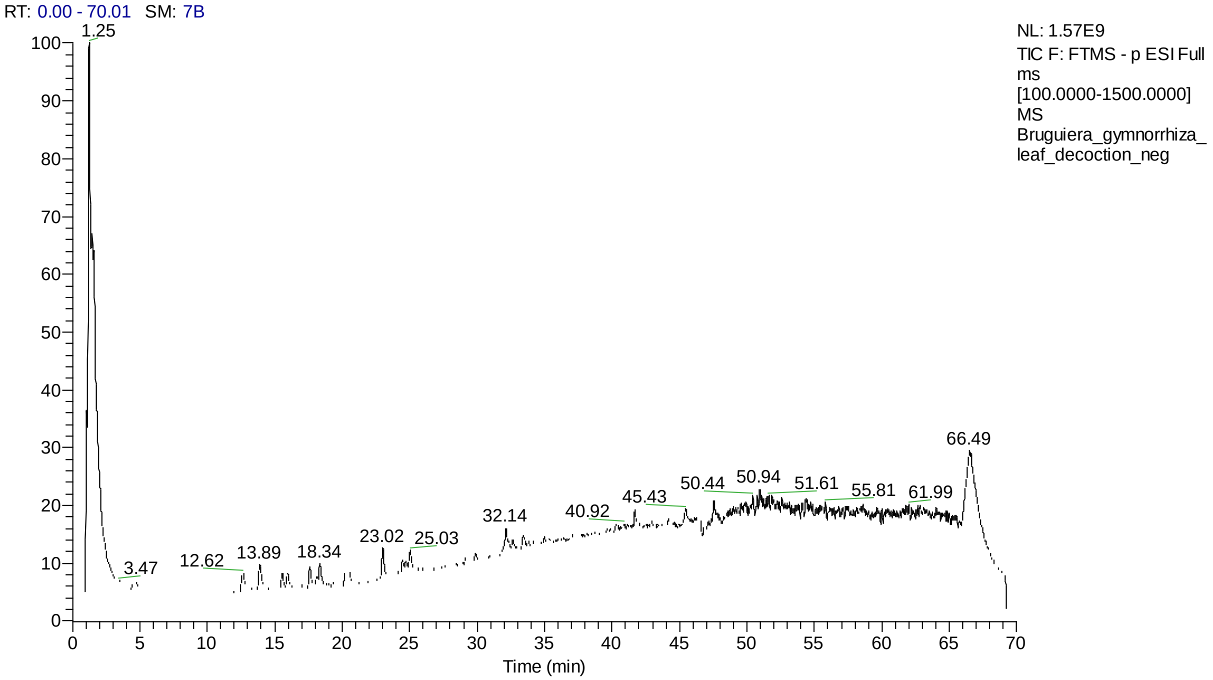

Figure S6. Bruguiera gymnorrhiza leaf decoction TIC negative.

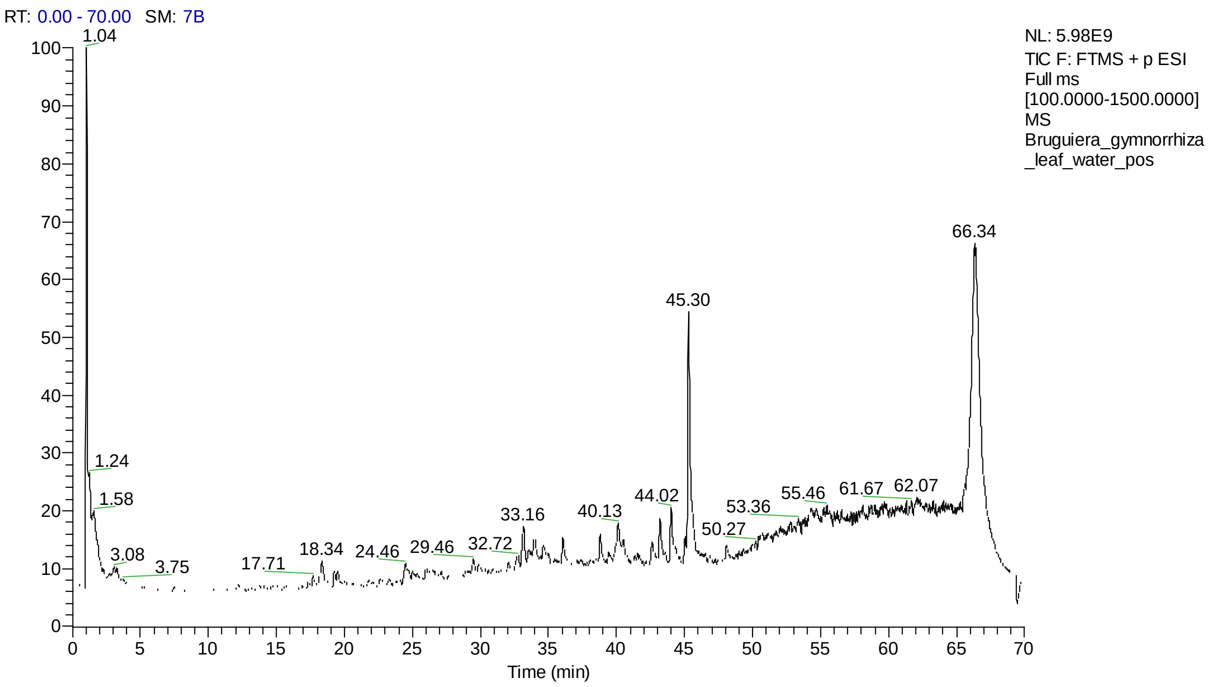

Figure S7. Bruguiera gymnorrhiza leaf water TIC positive.

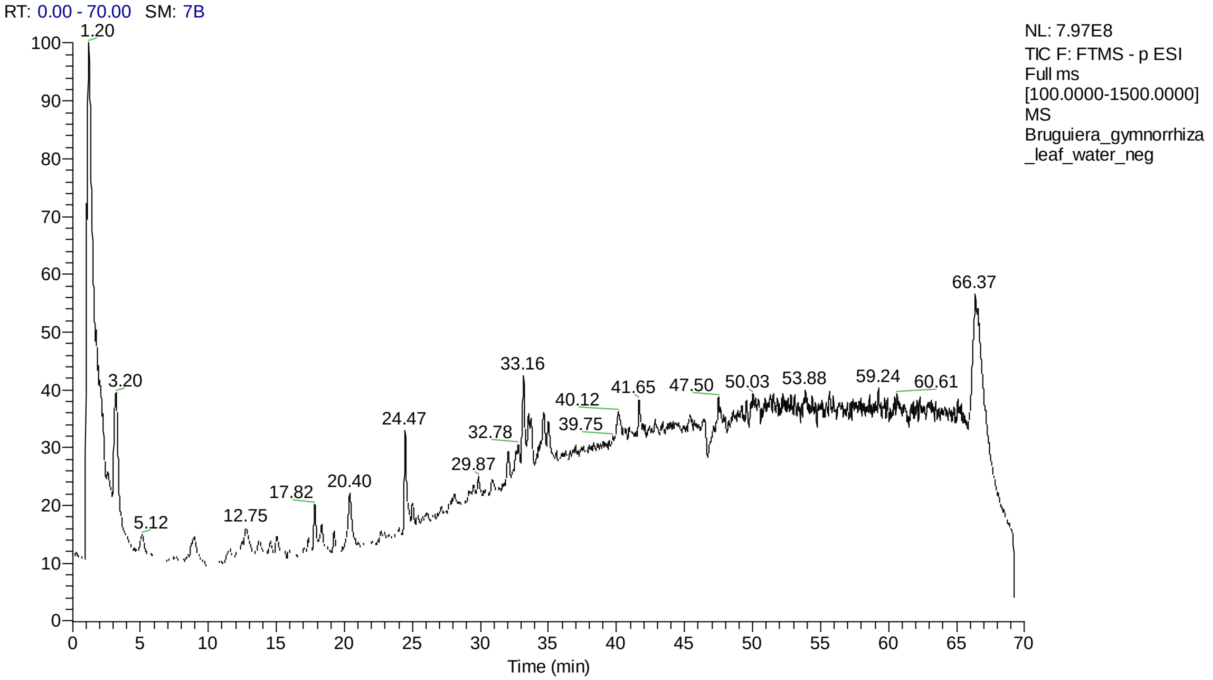

Figure S8. Bruguiera gymnorrhiza leaf water TIC negative.

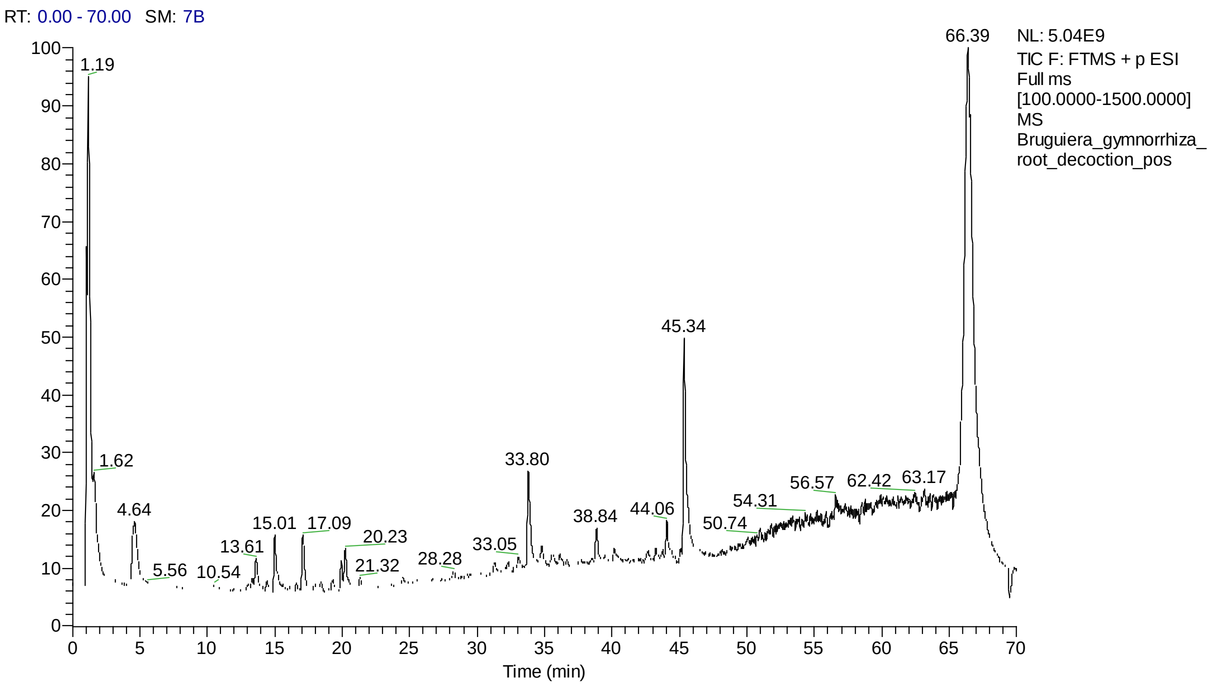

Figure S9. Bruguiera gymnorrhiza root decoction TIC positive.

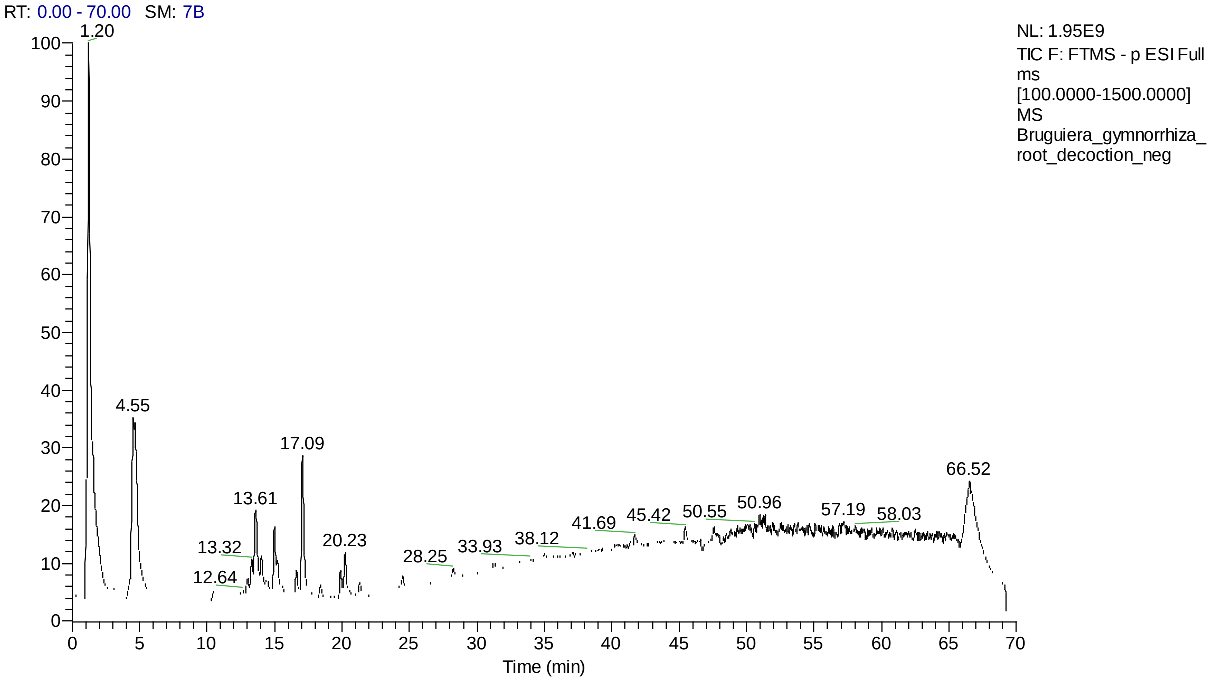

Figure S10. Bruguiera gymnorrhiza root decoction TIC negative.

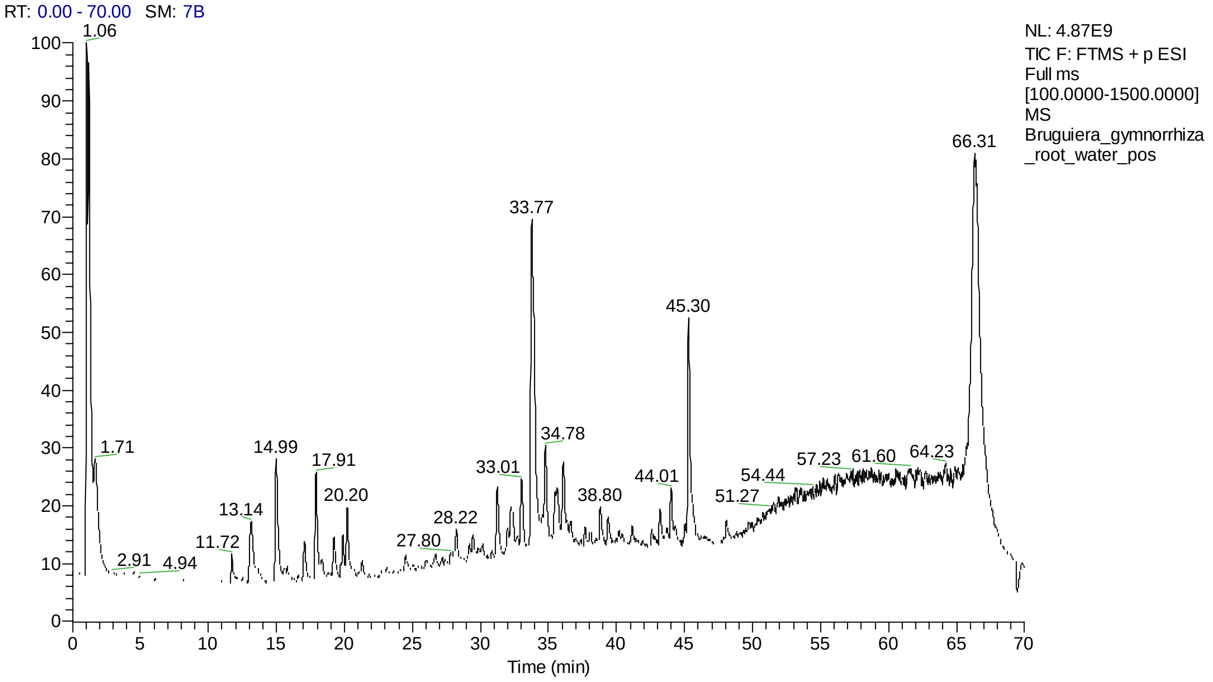

Figure S11. Bruguiera gymnorrhiza root water TIC positive.

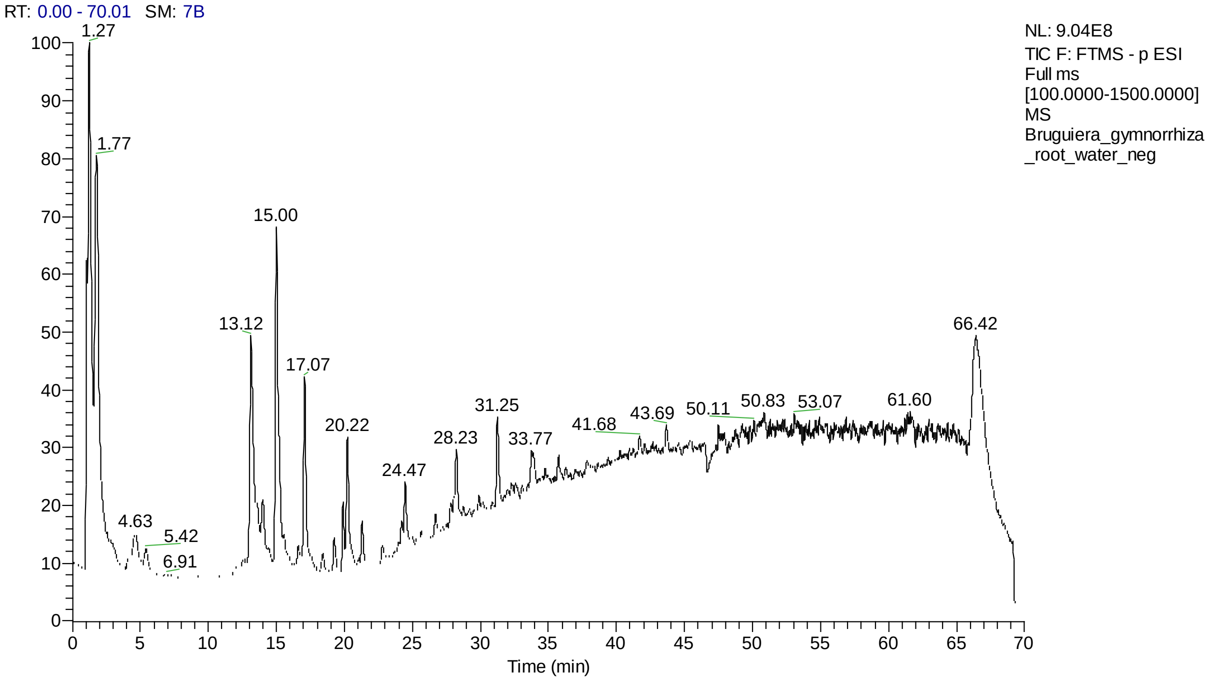

Figure S12. Bruguiera gymnorrhiza root water TIC negative.

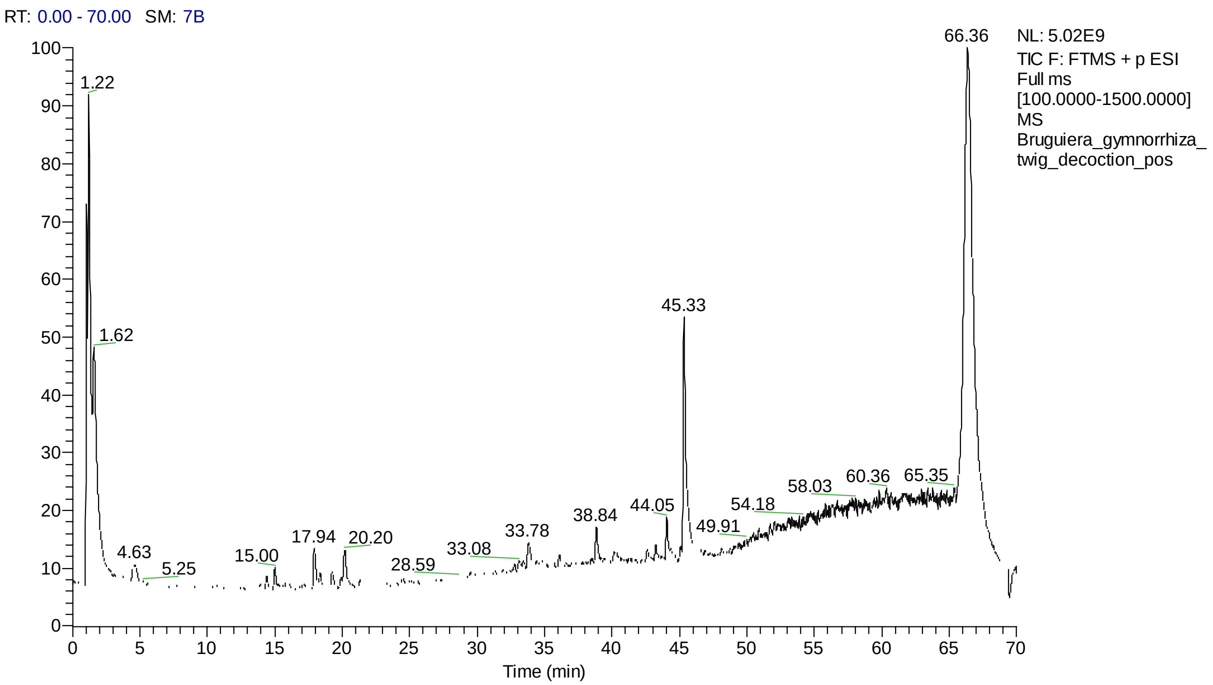

Figure S13. Bruguiera gymnorrhiza twig decoction TIC positive.

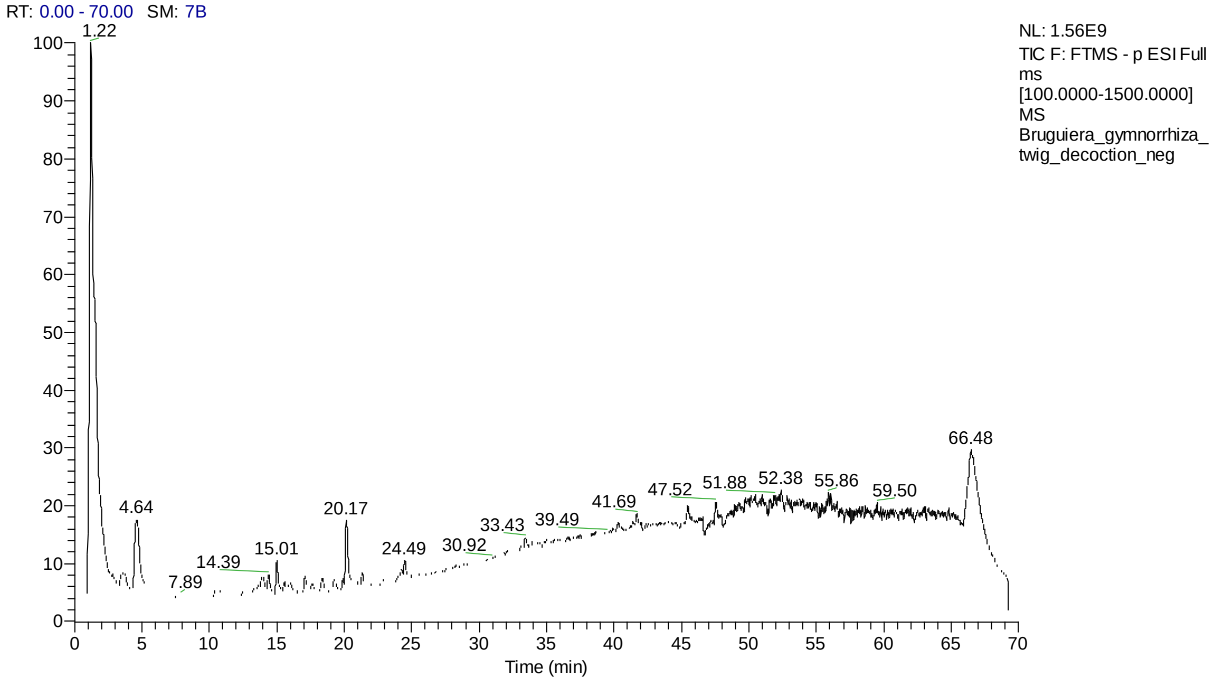

Figure S14. Bruguiera gymnorrhiza twig decoction TIC negative.

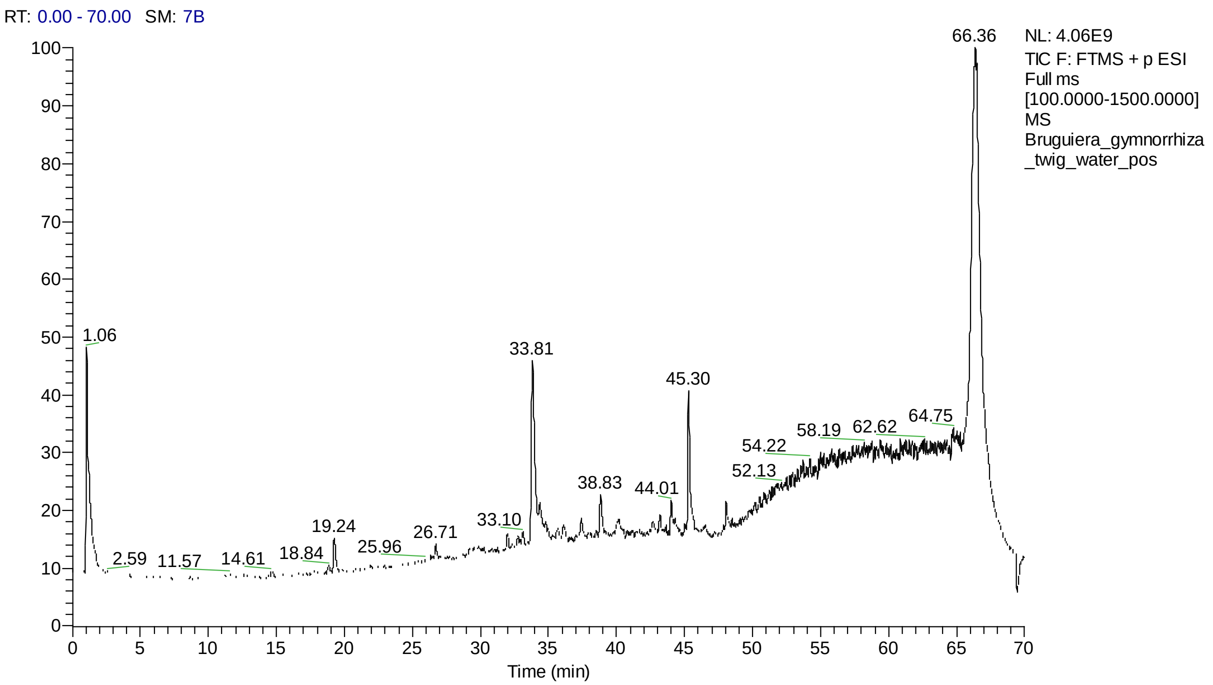

Figure S15. Bruguiera gymnorrhiza twig water TIC positive.

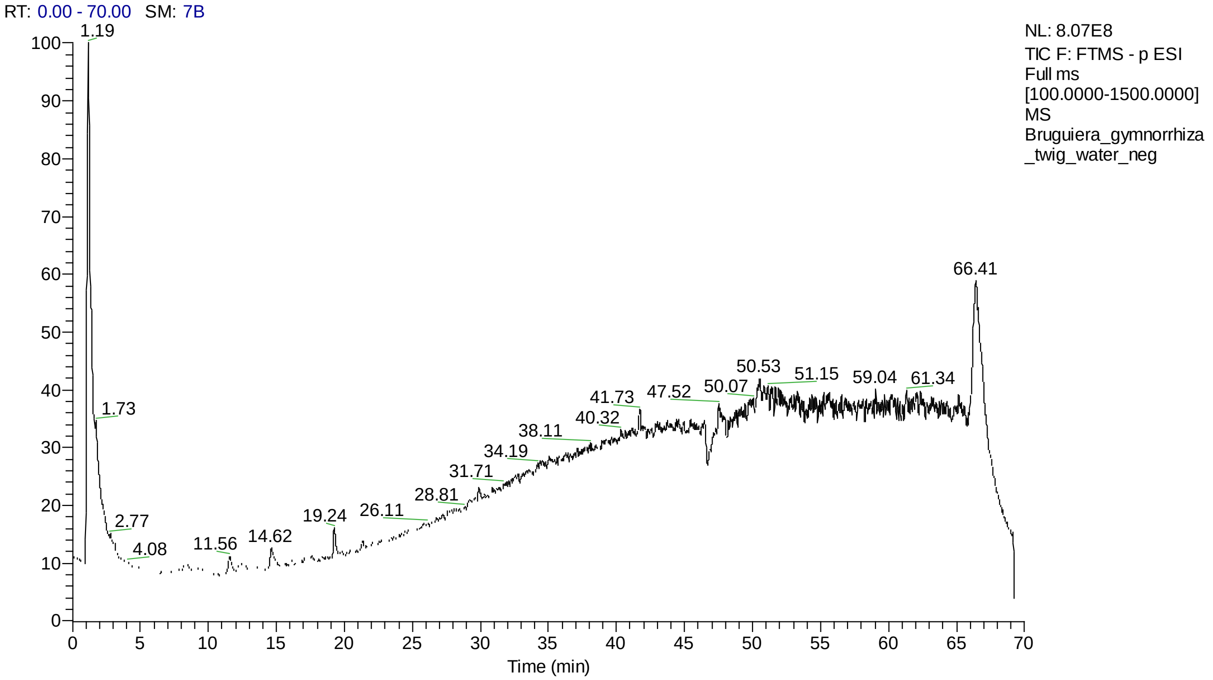

Figure S16. Bruguiera gymnorrhiza twig water TIC negative.
